# The RPL/RPS Gene Signature of Melanoma CTCs Associates with Brain Metastasis

**DOI:** 10.1158/2767-9764.CRC-22-0337

**Published:** 2022-11-16

**Authors:** Tetiana Y. Bowley, Irina V. Lagutina, Carol Francis, Sinduja Sivakumar, Reed G. Selwyn, Erik Taylor, Yan Guo, Bridget N. Fahy, Bernard Tawfik, Dario Marchetti

**Affiliations:** 1 Division of Molecular Medicine, Department of Internal Medicine, University of New Mexico Health Sciences Center, Albuquerque, New Mexico.; 2 Animal Models Shared Resource, The University of New Mexico Comprehensive Cancer Center, Albuquerque, New Mexico.; 3 Department of Radiology, University of New Mexico Health Sciences Center, Albuquerque, New Mexico.; 4 Division of Surgical Oncology and Palliative Medicine, University of New Mexico Comprehensive Cancer Center, Albuquerque, New Mexico.; 5 Division of Hematology and Oncology, Department of Internal Medicine, University of New Mexico Comprehensive Cancer Center, Albuquerque, New Mexico.

## Abstract

Melanoma brain metastasis (MBM) is linked to poor prognosis and low overall survival. We hypothesized that melanoma circulating tumor cells (CTC) possess a gene signature significantly expressed and associated with MBM. Employing a multipronged approach, we provide first-time evidence identifying a common CTC gene signature for ribosomal protein large/small subunits (RPL/RPS) which associate with MBM onset and progression. Experimental strategies involved capturing, transcriptional profiling, and interrogating CTCs, either directly isolated from blood of patients with melanoma at distinct stages of MBM progression or from CTC-driven MBM in experimental animals. Second, we developed the first MRI CTC-derived MBM xenograft model (MRI-MBM CDX) to discriminate MBM spatial and temporal growth, recreating MBM clinical presentation and progression. Third, we performed the comprehensive transcriptional profiling of MRI-MBM CDXs, along with longitudinal monitoring of CTCs from CDXs possessing and/or not possessing MBM. Our findings suggest that enhanced ribosomal protein content/ribogenesis may contribute to MBM onset. Because ribosome modifications drive tumor progression and metastatic development by remodeling CTC translational events, overexpression of the CTC RPL/RPS gene signature could be implicated in MBM development. Collectively, this study provides important insights for relevance of the CTC RPL/RPS gene signature in MBM, and identify potential targets for therapeutic intervention to improve patient care for patients with melanoma diagnosed with or at high risk of developing MBM.

**Significance:**

This study reports first evidence of RPL/RPS gene signature driving melanoma brain metastasis. Complex multilevel approach was performed to identify MBM signature and confirm its relevance to clinical settings. Novel MRI CTC-derived MBM mouse xenograft was established to monitor MBM spatial and temporal development and progression.

## Introduction

Melanoma is the most aggressive and lethal skin cancer, and the one with highest propensity to generate brain metastasis (MBM; refs. 1–3). MBM is diagnosed clinically in up to 60% of patients with metastatic melanoma and in up to 80% of patients at autopsy. A poor prognosis (4–6 months survival), and extreme deterioration in quality of life have been reported for patients with MBM (1, 4–8). The high mortality rate of patients with MBM is linked to brain tumor expansion, hemorrhage, increased intracranial and extracranial pressure (7, 9). At time of autopsy, the tumor mass is often larger than clinical imaging suggests (9). Local therapies include resection of a single MBM lesion, if surgically accessible, and radiation (9, 10). Other therapeutic interventions include systemic therapies, such as targeted or immune-based therapies (9, 11). While checkpoint inhibitors have yielded some promising results treating patients with MBM (1, 6, 7, 12), clinical activity in the brain is significantly less than in extracranial metastasis.

Metastasis is a complex multistep process enabling the spread of tumor cells from a primary tumor to distant organs, resulting in poor prognosis and high morbidity (9, 13). Specifically, melanoma cells have the capability to metastasize to most organs, with most common sites being the lungs, skin, liver, and brain (1). The brain microenvironment represents a unique niche due to the selective semipermeable blood–brain barrier, high nutrient and energy consumption, and immune privilege (9, 14). Circulating tumor cells (CTC) are “seeds” of fatal metastatic disease and smallest functional units of cancer. CTCs disseminate from primary and/or metastatic tumors into vasculature and initiate tumor development at distant organs (15–17). Only a small fraction of CTCs can successfully develop into metastasis/MBM, due to the harsh physical, oxidative, and other microenvironmental stresses they encounter in blood (18, 19). Extensive reports have also demonstrated that CTC dissemination occurs early and that CTCs migrate to distant organs where they can initiate metastasis or remain dormant (16, 20). Importantly, cancer progression and clinical outcomes of patients with melanoma directly correlate with numbers of CTCs in the bloodstream (21).

Interestingly, recent studies have identified a link between abnormal ribosome biogenesis and increased tumor burden (22–25). For example, a study demonstrated that augmented expression of the ribosomal large-subunit protein 15 (RPL15) in breast cancer CTCs triggered massive metastatic spread and induced the translation of other ribosomal subunits proteins (24). Accordingly, enhanced expression of ribosomal proteins result in ribosomopathies associated with metastatic development and progression (22, 23).

We hypothesized that the comprehensive multilevel characterization of melanoma CTCs/Lin− cells isolated from patients (FACS sorted for absence of normal circulatory cells and Lin+ cells; ref. 26) and/or CTC xenografts with and/or without MBM can be critical to identify novel biomarkers and to evaluate effective therapies targeting and/or preventing MBM. Specifically, we postulated the existence of a common CTC genetic signature uniquely associated with MBM onset and its progression over time. We evaluated this by performing complex multilevel analyses of CTCs correlating with MBM progression in patients with melanoma, additive to employing a novel MBM CTC xenograft model (MBM-CDX). Furthermore, we used MRI to detect the spatial and temporal progression of MBM in a newly developed preclinical model (MRI-MBM CDX).

Here, we report the identification of the CTC RPL/RPS gene signature of MBM which was found to be common in CTCs characterized from all MBM samples analyzed, either from patients or xenograft models [the term “RPL” stands for 60S or large ribosomal subunit while “RPS” stands for 40S or small ribosomal subunit (the 40S and 60S subunits comprise the 80S ribosomal particle which initiates and regulates translation)]. Moreover, by employing the MRI-MBM CDX model, we demonstrate that the CTC RPL/RPS gene signature was significantly expressed in CTCs from all samples analyzed either spatially or longitudinally, and was significantly associated with MBM onset and progression. The discovery of enhanced expression of the CTC RPL/RPS gene signature of MBM sets the stage for the development of putative RPL/RPS therapeutic targets to improve MBM patient care.

## Materials and Methods

### Patient Blood Collection and Processing

Patients diagnosed with primary or metastatic melanoma were enrolled according to protocols approved by the Institutional Review Board at UNM Health Sciences Center (UNM-HSC), Albuquerque, NM. All patients’ blood samples were collected after receiving informed written consent, according to the principles of Declaration of Helsinki. Clinical details of each patient included in the study are provided in Supplementary Table S1. Peripheral blood (12–18 mL) was collected either in CellSave (Menarini Silicon Biosystems, Inc.), or in sodium-ethylenediamine tetraacetic acid (EDTA) tubes under aseptic conditions. Blood collection was performed at the middle of vein puncture as part of patients’ routine clinical care. Following blood collection, samples were sent immediately to the laboratory for isolation and analysis of CTCs. All blood specimens were analyzed within 24 hours following blood draw.

### CellSearch CTC Enumeration

CTCs positive for the human melanoma biomarker Mel-A (Mel-A+ CTCs) were captured and quantified by the CellSearch platform (Menarini Silicon Biosystems, Inc.), following manufacturer's guidelines. Samples (7.5 mL) were processed using CellTracks and the CellSearch melanoma CTC kit. CellSearch-captured CTCs are defined as MEL-PE^+^/DAPI^+^/CD45^−^ cells (27, 28). Peripheral blood (7.5 mL) from healthy donors was used as negative control and subjected to the same process. In addition, the human melanoma CTC-derived clonal lines (70W-SM3 cells) were spiked at different concentrations in 7.5 mL of healthy donor blood as positive control. The automated CellBrowser software was used to visualize and quantify CellSearch melanoma CTCs.

### Peripheral Blood Mononuclear Cell Isolation and CTC Enrichment by FACS

Peripheral blood mononuclear cells (PBMC) were isolated by an established procedure (27, 29). Briefly, patients’ blood was lysed with red blood cell lysis buffer (BioLegend, catalog no. 420302), and washed twice with PBS with 5 mmol/L EDTA (USB, catalog no. 15694). PBMCs were isolated and quantified by the Countess II cell counter (Thermo Fisher Scientific). Following cell blocking with Fc block (BioLegend, catalog no. 422302), PBMCs were stained for fluorescence labeling with FITC-CD45 (BioLegend, catalog no. 304038), FITC-CD34 (BioLegend, catalog no. 343504), FITC-CD73 (BioLegend, catalog no. 344016), FITC-CD90 (BioLegend, catalog no. 328108), FITC-CD105 (BioLegend, catalog no. 323204), Pacific Blue–conjugated CD235 (BioLegend, catalog no. 306612). Processed cells were then sorted using a iCyt SY3200 cell sorter (Sony Inc.) to separate Lineage-negative (Lin−) and Lineage-positive (Lin+) cell populations. FITC-positive cells were sorted into the Lin+ fraction, while the Lin− fraction consisted of cells negative for all fluorescent biomarkers indicative of normal cell lineage. Briefly, FACS gating employed the depletion of dead cells (DAPI^−^), followed by the isolation and elimination of leukocytes (CD45^+^), erythrocytes (CD235^+^), endothelial cells (CD34^+^), and mesenchymal stromal cells (CD73^+^/CD90^+^/CD105^+^; refs. 27–29). CD235-positive cells were eliminated from downstream analysis. Data generated by FACS were analyzed by FlowJo V10 program, as described previously (27, 29).

### RNA Sequencing

RNA was isolated from Lin− and Lin+ fractions (25–50 × 10^3^ cells, respectively) after FACS. RNA extraction was performed using a miRNA Isolation kit (Qiagen Inc., catalog no. 74004). RNA from matching Lin− and Lin+ fractions were compared with RNA from PBMCs of healthy donors (negative controls). RNA analysis, cDNA amplification, and library preparation were performed using the human microarray platform (SMARTer Universal Low Input RNA kit for sequencing (Clontech, catalog no. 634946). The Ion Plus Fragment Library kit (Thermo Fisher Scientific, catalog no. 4471252) was used for fragmented RNA, as reported previously (30–32). The Ion Proton S5/XL platform (Thermo Fisher Scientific) was used for sequencing at the Analytical and Translational Genomics Shared Resource Core at the University of New Mexico Comprehensive Cancer Center (UNM-CCC).

### Bioinformatics and Biostatistical Analyses

RNA sequencing (RNA-seq) analyses were aligned using tmap (v5.10.11) to a BED file that contained nonoverlapping exon regions from the UCSC genome browser (GRCh38/hg38). HTSeq (v0.11.1) was used to quantify exon counts (26, 33). The gene-level counts were generated by averaging counts across exons. Normalization of the library size and differential analysis were carried using edgeR (26, 34). Heatmap and cluster analysis were conducted using Heatmap3. Pathway enrichment analyses were executed using clusterProfiler, Pathview, and topGO software programs (26, 34). Data generated by pathway discrimination analyses were analyzed by the Reactome pathway database, as described previously (35).

### Cell Culture

Highly brain-metastatic melanoma CTC-derived clonal cells (70W-SM3; generated in Dr. Marchetti's laboratory; ref. 27) or the human melanoma MeWo line (ATCC; catalog no. HTB-65) were stored in liquid nitrogen and freshly recovered prior to use. Cells were maintained at 37°C in a humidified 5% CO_2_ incubator in DMEM nutrient mixture F-12 (DMEM/F12; Gibco, catalog no. 11320033), supplemented with 10% FBS (Gibco, catalog no. A4766801). Cells were grown using ultra-low attachment plates (Corning, catalog no. CLS3471), routinely tested for *Mycoplasma* using *Mycolpasma* Detection Assay (MycoAlert, Lonza) every 20 passages, and were only used at low-passage number (lower than 30 passages). PCR-based assay for authentication of cell lines was performed routinely. Luciferase-tagged 70W-SM3 cells were acquired using procedures reported previously (36). Prior to use, cells were checked for phenotypic changes using microscopy.

### CTC/CTC Cluster Capturing

Peripheral blood (7.5 mL) was collected from patients in EDTA-coated tubes, and loaded onto the CTC Parsortix microfluidic chip (8 μm) within 1 hour of blood draw. Samples were analyzed employing the CTC filtration and/or microfluidic Parsortix PR1 instrument (Angle Europe Ltd.), and 6.5 μmol/L cartridges (Angle PLC). Following cassette priming, blood went through the cassette capturing single CTCs and CTC clusters based upon their size and deformability. To analyze captured CTC/CTC clusters, cells were either harvested and subjected to RNA isolation, or immunostained inside the Parsortix separation cassette, according to manufacturer's instructions (28). CTCs were defined and enumerated based upon positivity for human Mel-A (Alexa Fluor 594-tagged, Santa Cruz Biotechnology, catalog no. sc-20032), and human DAPI (Thermo Fisher Scientific, catalog no. D3571) staining, however negative for human CD45 (FITC-tagged, BioLegend, catalog no.103108) staining. Parsortix-captured cells displaying the human Mel-A^+^/DAPI^+^/CD45^−^ phenotype with a round and intact morphology were designated as CTCs. Confocal microscopy was performed for CTC visualization and enumeration of CTC/CTC clusters using Zeiss LSM800 microscope (10–40× magnification) and ZEN system software (Carl Zeiss Microscopy).

### CDXs

All *in vivo* studies were performed according to the approved Institutional Animal Care and Use Committee protocol. Animal studies were carried out using 6 to 12 weeks old immunodeficient NOD.Cg-Prkdcscid Il2rgtm1 Wjl/SzJ (NSG) mice (Jackson Labs). Mice were given 50 μL (4 mg/mL) low-molecular weight heparin intravenously (retro-orbital or tail vein) 10 minutes prior to intracardiac injection of MBM CTC-derived clone (70W-SM3-Luc2 cells) to prevent thromboembolism in mice (37). For intracardiac injections, mice were anesthetized with isoflurane (2.5%, 1 L/minute O_2_ flow), placed in dorsal recumbency, and injected into the left ventricle (5.0 × 10^5^ cells in 50 μL of PBS) using a sterile 0.5-mL U-100 insulin syringe with a 29Gx1/2″ needle (Beckton Dickinson, catalog no. 58324702). The injection site was confirmed as intracardiac by blood backflow into the syringe prior to injection. Animals were then monitored on a daily basis for changes in health status (rapid weight loss, distress, difficulty with breathing or ambulation, impaired mobility, seizures, ruffled coat, difficulty in obtaining food or water, etc.). For CTC capture and enumeration in animals over time, blood (100–150 μL) was collected from mouse retro-orbital sinus using EDTA-coated glass Pasteur pipette into a MiniCollect tube (Greiner Bio-One, catalog no. K3E K3EDTA). Prior to blood collection, mice were anesthetized with isoflurane (2.5%, 1 L/minute O_2_ flow). Tumor development was monitored weekly by Xenogen IVIS Spectrum animal imager (PerkinElmer), with acquisition of both two-dimensional and three-dimensional (3D) optical tomography using Living Image Software program (PerkinElmer). For *in vivo* assessment of tumor burden, luciferin (150 mg/kg) was administered intraperitoneally into a mouse 10 minutes prior to imaging. At the end of the study, mice were sacrificed, necropsied, and weighed, and blood (0.6–1.0 mL) was collected via retro-orbital injection into an EDTA-containing MiniCollect tube (Greiner Bio-One, catalog no. K3E K3EDTA). Mice were kept under isoflurane anesthesia (5%, 1 L/minute O_2_ flow), until opening the chest cavity. Liver, lungs, and brain organs were snap-frozen in Tissue-Tek OCT compound (Sakura Finetek USA Inc., catalog no. 4583). Spleen, sternum, femur, and skull-cap tissues were fixed in 10% neutral buffered formalin for pathologic evaluation. Because most melanoma cells produce melanin, melanoma metastasis was visually detected as brown-to-black pigmented regions (38).

### MRI

Animals whose MBM was detected 24 hour postinjection of CTC-derived clonal cells (70W-SM3) were selected for MRI. MRI was conducted biweekly using the advanced Bruker 7 Tesla PET/MRI instrument (Bruker Inc.) to detect and monitor melanoma progression in the brain. The first MRI session was 3 days postinjection and considered day 0 of MRI studies. MRI was used to assess the presence of tumors in Gadolinium contrast-enhanced (CE) T1-weighted (T1W) and brain structures in T2-weighted (T2W) MRI. Image resolution for T1W and T2W MRI was 100 × 100 × 500 μm^3^. The skull stripping technique was performed on the T2W MRI sequence to remove extrameningeal tissues from brain images of the whole head and to better visualize tumors. T2-weighted images were acquired with a fast spin-echo sequence rapid acquisition with relaxation enhancement with repetition time (TR)/echo time (TE)  = 5,000 ms/30 ms, field of view (FOV)  =  15 mm ×  15 mm, slice thickness  =  0.5 mm, interslice distance  =  0.5 mm, number of slices  =  30, matrix  = 150  ×  150, number of average  =  1. T1-weighted images were acquired with a 3D fast low angle shot with TR/TE  =  20 ms/5 ms, FOV  = 15 mm ×  15 mm × 15 mm, slice thickness  =  0.5 mm, interslice distance  =  0.5 mm, number of slices  =  30, matrix  =  150  ×  150, number of average  =  9. Fast T1 maps were developed using inversion recovery (IR) based T1_EPI (echo planar imaging) with RT/TE  =  3,000 ms/10.2 ms, FOV  =  15 mm ×  15 mm × 15 mm, slice thickness  =  0.5 mm, interslice distance  =  0.5 mm, number of slices  =  30, matrix  =  100  ×  100, number of average  =  1, EPI segments = 8, automatic ghost correction = on, IR offset = 20, IR Spacing = 160, IR points = 16 (39, 40).

Prior to MRI, mice were given 100 μL (3.89 mL/kg) of contrast agent MultiHance gadobenate dimeglumine (Bracco Diagnostics Inc, catalog no. SP9002A) intravenously (retro-orbital or tail vein) to enhance tumor visualization. Contrast agent was injected right before placing the animal into the MRI scanner. The mouse was positioned in a dedicated holder and placed in the isocenter of the 7T MRI scanner (Bruker Biospin MRI), which was equipped with a 30 cm bore, a 20 cm gradient with the strength of 660 mT/m and shim systems (Bruker Biospin MRI). To obtain a good signal-to-noise ratio, a small bore linear RF coil (inner diameter  =  72 mm), and a phased-array surface coil were employed for signal excitation and detection, respectively. During MRI experiments, mice were anaesthetized with 1–1.5% isoflurane (Phonenix, Clipper Distributing Company) by mechanical ventilation. A monitoring system of physiologic parameter (SA Instruments, Inc) enabled the visualization of the respiratory cycle.

### MRI Analyses and Statistical Validation

MRI analyses were performed by the Radiology Department at UNM-HSC by one of the co-authors (E. Taylor). Images were organized by scan date and subject number, followed by whole brain bias field correction using the Advanced Normalization Tools software in Python (ANTsPy; Python Software Foundation; ref. 41). CE-T1W MRI was analyzed by 3D Slicer software (Linux, version 4.11.20210226). Brain tumors were semimanually segmented using the level tracing method for tumor volume measurement (41). T2W MRI was skull-striped (SS) by a deep learning technique with U-Net followed by manual correction of the SS image in 3D slicer. Brain atlas with 62 regions structures including frontal lobe (FL), parieto-temporal lobe (PTL), and other major brain regions (42) was spatially normalized to T2W images in ANTsPy by rigid, affine, and a deformable registration for each individual subject and timepoint was carried out. Total brain tumor volume and regional brain tumor volume were then calculated from segmented CE-T1W MRI labeled with the brain atlas. Brain tumors were counted using scikit-image (43) measure label tool to assign all 3D connected regions with a unique integer value in Python. Brain atlas labels were then referenced to assign each tumor >10 voxels to a brain region of interest.

### Data Availability

NCBI SRA database BioProject accession number PRJNA866169.

## Results

Patient CTCs exhibit extensive heterogeneity in their cell surface biomarkers (27, 34, 44). The absence of a universal CTC biomarker is particularly valid in melanoma (27), creating a challenge for the detection and capture of the entire spectrum of CTC subsets present and implicated in melanoma carcinogenesis and metastasis (27, 44, 45). Multiple CTC platforms have been used to detect and isolate melanoma CTCs, including CellSearch (46–48). CellSearch is the only FDA-cleared platform for CTC isolation, visualization, and interrogation [FDA clearance is however applicable only for metastatic breast, prostate, and colorectal cancers, not melanoma (17, 27, 45)]. Specifically, the melanoma CellSearch CTC kit uses MEL-PE (CD146) biomarker to capture CTCs. Captured CTCs are then detected, visualized, and enumerated via automated CellBrowser software. Accordingly, a consequence of melanoma CTC heterogeneity is inability of the CellSearch assay to isolate and study the entire CTC spectrum beyond MEL-PE^+^/DAPI^+^/CD45^−^ cells.

**FIGURE 1:**
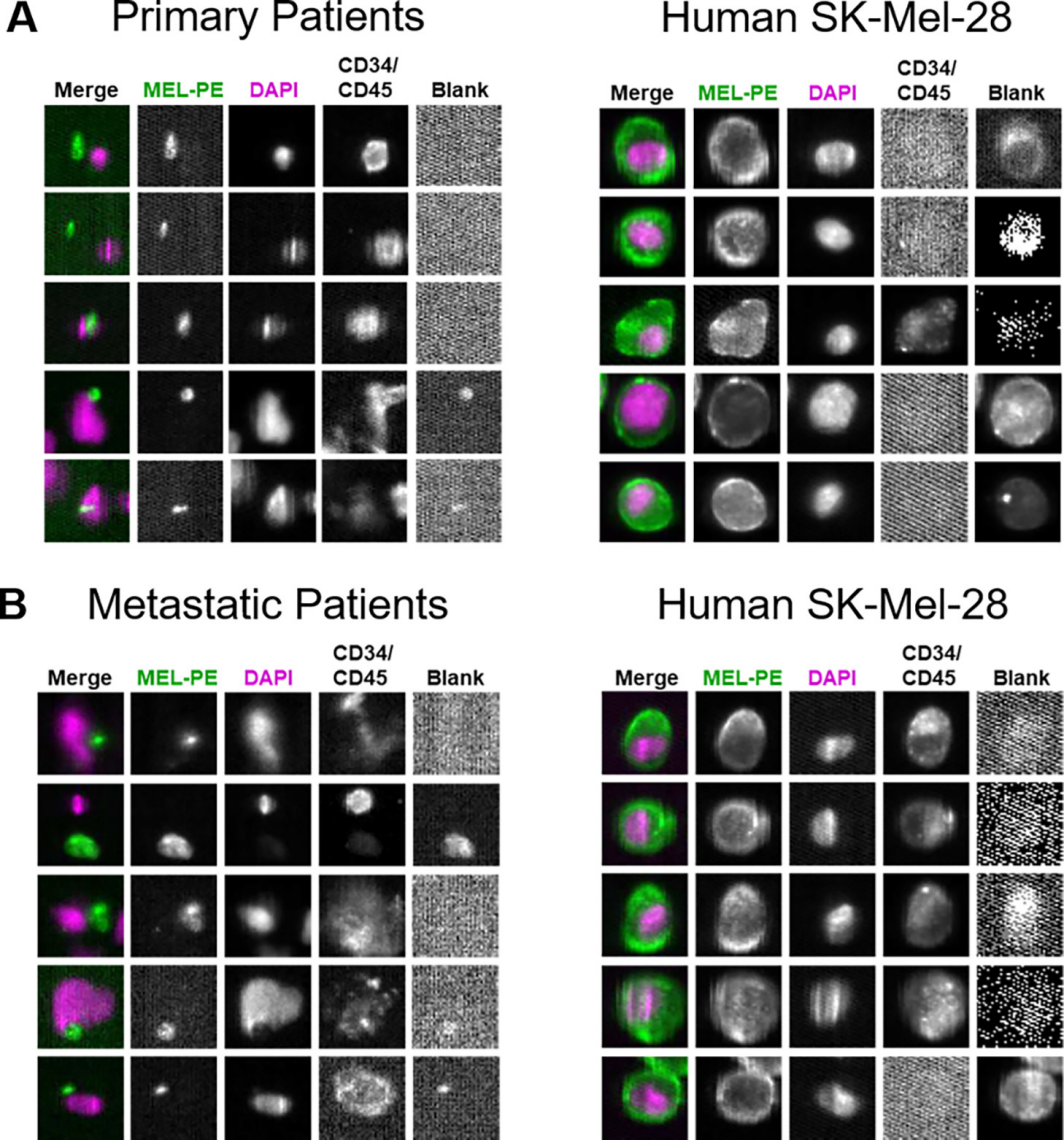
The capture, visualization, and enumeration of melanoma CTCs (MEL-PE^+^/DAPI^+^/CD34/CD45^−^ cells) from patients’ blood using the CellSearch platform and CellSearch melanoma assay (Menarini Silicon Biosystems, Inc.). Peripheral blood (7.5 mL) was obtained from patients with primary (**A**) and metastatic (**B**) melanoma, and analyzed by CellSearch. No CTCs were detected in these patient samples as MEL-PE^+^/DAPI^+^/CD34/CD45^−^ cells, according to CellSearch analyses. Cells from the human melanoma SK-Mel28 line (CellSearch melanoma CTC kit) were analyzed in parallel as positive control for (right). Displayed are the original CellSearch images using CellBrowser software (10x magnification).

As first step, we collected and processed peripheral blood from patients with primary or metastatic melanoma by CellSearch. No CTCs could be detected by the CellSearch platform in any of these analyses (Fig. 1A and B). To confirm validity of these results, human melanoma cells (SK-Mel-28 line) embedded within the CellSearch melanoma CTC assay and run in parallel to patient samples showed a high number of CTCs being captured (positive control; Fig. 1). Healthy donors’ blood was analyzed via CellSearch with negligible results (negative control; Supplementary Fig. S1A). Similar CellSearch analyses using the CTC-derived melanoma clone (70W-SM3 cells) spiked in healthy donors’ blood at different concentrations displayed consistent CTC capturing and/or visualization (Supplementary Fig. S1B). These findings suggest that CellSearch cannot detect melanoma CTCs in patient samples based solely on the presence of the MEL-PE (CD146) biomarker selection.

Consequently, we selected a multilevel approach to characterize CTCs and evaluate a CTC-associated gene signature responsible for MBM onset. To discriminate gene expression differences among CTC populations in patients with primary and metastatic melanoma, we implemented multiparametric flow cytometry (FACS) to deplete circulatory normal cell lineages (Lin+ or LinP cells) from peripheral blood of patients, thus selecting a cell population of neoplastic origin (referred as Lin− or LinN cells here and onward; ref. 27).

**FIGURE 2:**
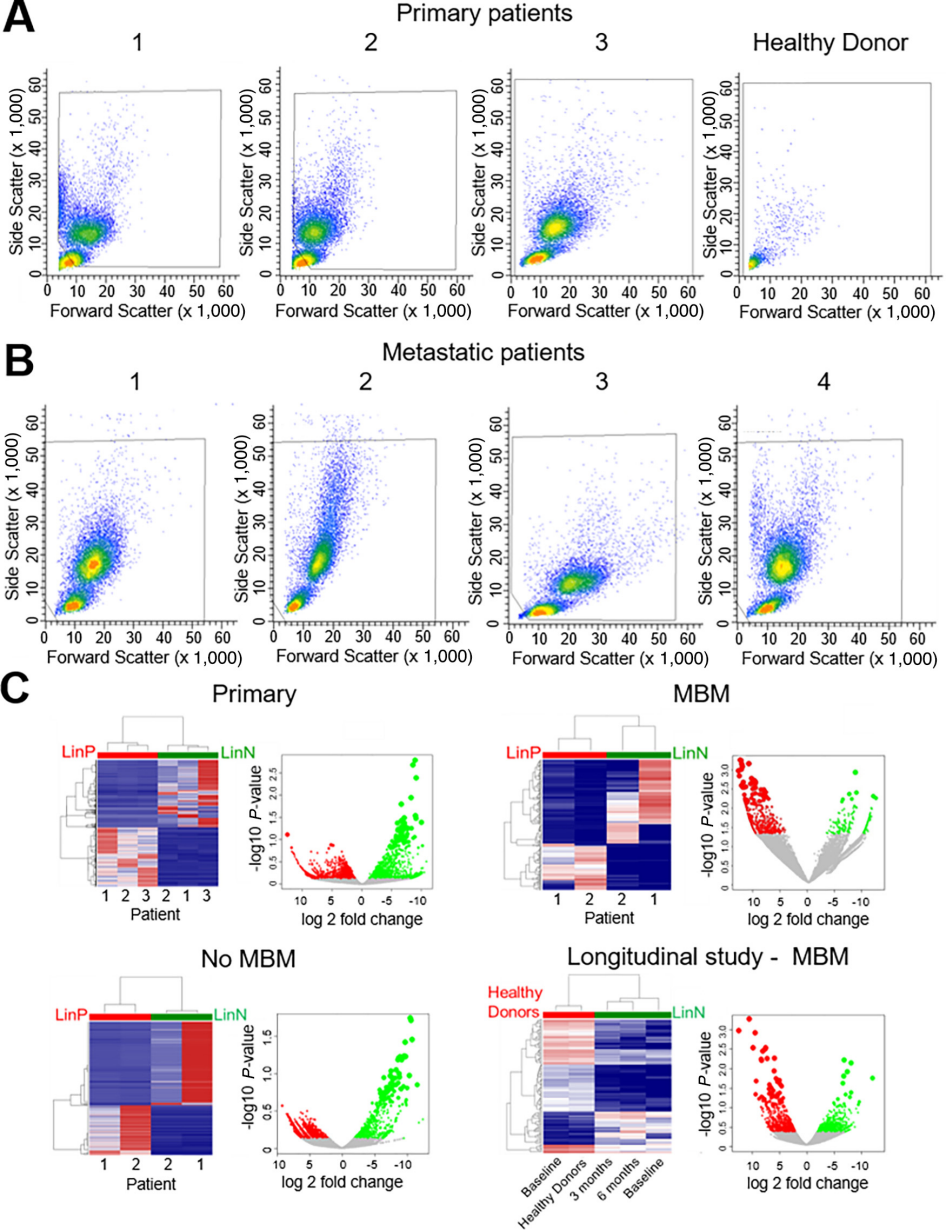
Multiparametric flow cytometry gating for the isolation of viable Lin-negative/CTC-enriched populations from a number of independent patients with primary (**A**), and metastatic (**B**) melanoma. Enrichment of Lin-negative cell populations (CD45^−^/CD34^−^/CD73^−^/CD90^−^/CD105/CD235^−^ cells) was performed, as reported previously (27). The same multiparametric FACS procedure was applied to healthy donor blood, showing no presence of Lin-negative cell population (negative control). **C,** Transcriptional profiling detailing discordance among Lin-positive (LinP) versus Lin-negative (LinN) cell populations, and LinN heterogeneity from independent patients with primary or metastatic (diagnosed with or without MBM) melanoma. Hierarchical clustering of gene expression profiling showing significant differences between the LinN (green) and LinP (red) cell populations isolated from primary, MBM, metastatic patients without MBM diagnosis (No MBM), and LinN cell populations isolated over time (0, 3, 6 months longitudinal collection) from a patient diagnosed with MBM, and compared with LinP cells isolated from blood of healthy donors, respectively. Each LinN/LinP population is patient paired (same patient). Scatter plots show global gene expression of LinN cell populations with significant log2 fold change (green dots), compared with LinP/healthy donor cell populations (red dots). See “Materials and Methods” for experimental details.

Next, we performed RNA-seq on FACS-sorted Lin−/Lin+ cells to assess whether Lin− cell populations isolated from primary melanoma without clinical evidence of metastasis or Lin− cells isolated from patients with metastatic melanoma regardless of MBM could reflect the evolution of melanoma in the blood (Fig. 2). Normal blood served as negative control (Fig. 2A). We carried out the negative depletion strategy to isolate CTC-enriched Lin− fraction from the Lin+ cell population for every sample. Analyses of Lin−/Lin+ samples from patients with and/or without MBM were performed in parallel to compare Lin− gene signatures from patients (Fig. 2A and B). Not all metastatic patients exhibited brain metastasis. The metastatic sites for each patient are presented in Supplementary Table S1. Specifically, patients with MBM had brain metastasis, while patients with No MBM had metastasis to distant organs, but not to the brain. Hierarchical clustering not only showed the distinction among Lin−/Lin+ cell transcriptomes, but also significant differences among Lin− cell fractions at distinct stages of melanoma progression to MBM, reflecting CTC/Lin− heterogeneity (Fig. 2C). Of note, an extensive (0–6 months) longitudinal investigation of Lin− transcriptomics was performed in a patient with MBM to evaluate gene expression signatures relatable to MBM progression within the same individual (Fig. 2C). This patient with known MBM underwent treatment (nivolumab) and periodic MRIs which did not show any new or progressive intracranial metastatic lesions.

RNA-seq analyses of these samples were performed, and unsupervised hierarchical clustering revealed distinct transcriptomic profiling of the CTC-enriched Lin− fraction in all four analyses (Fig. 2C). Furthermore, detailed transcriptomic analyses of the Lin− fraction of patients with MBM and the longitudinal monitoring of an individual patient with MBM were integrated with MBM mouse transcriptomics data to yield common upregulated and/or downregulated genes, and to identify common gene signatures using a four-level discrimination approach discussed below.

### Spatial and Temporal Divergence of CTC-MBM Transcriptomic Signatures

**FIGURE 3:**
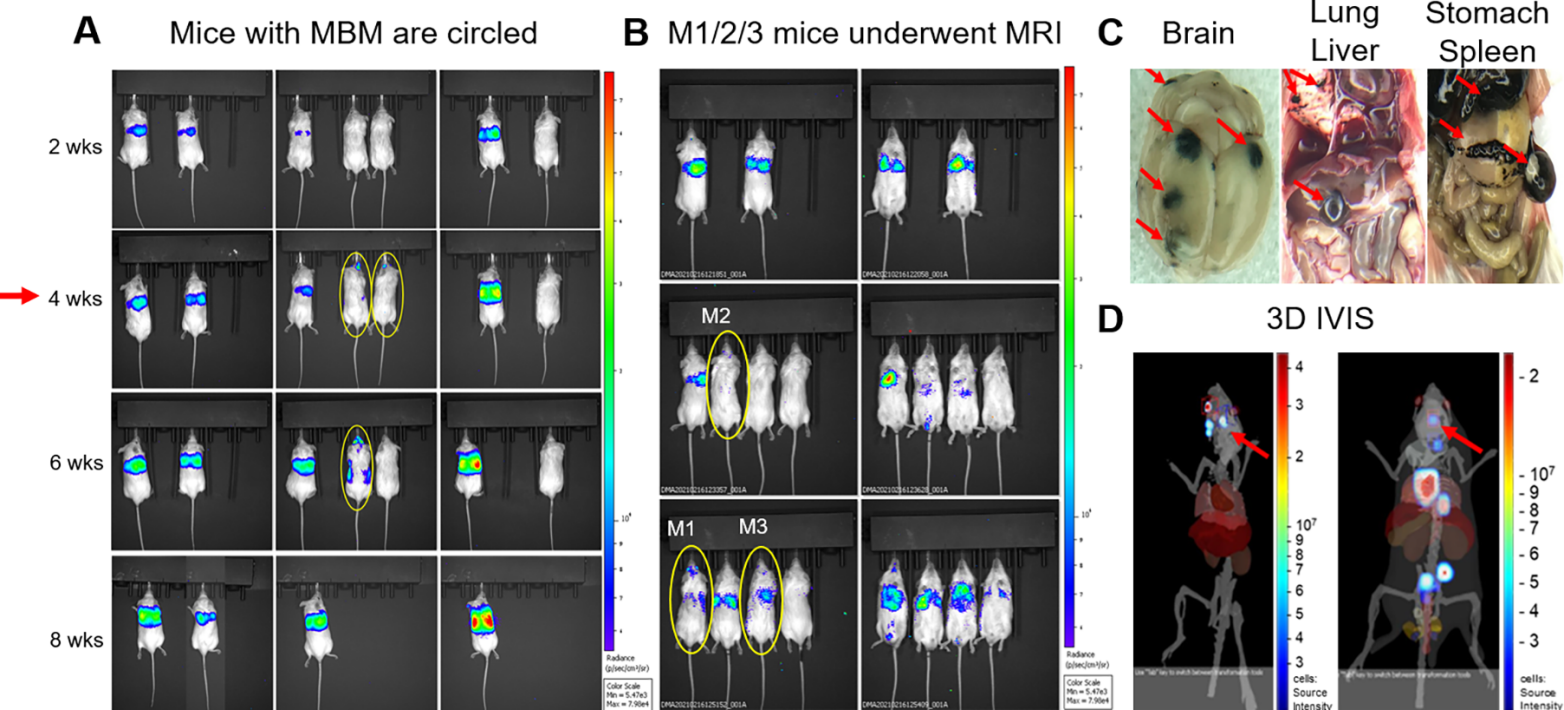
The generation of the MBM CTC xenograft model (MBM CDX). **A,** Immunodeficient (NSG) mice were injected intracardiacally with the MBM CTC-derived clone (5.0 × 10E5 70W-SM3-Luc2 cells), and subsequently imaged by IVIS to evaluate MBM onset with parallel pathologic examination. Consistent MBM (mice with yellow circles) was observed at 4 weeks postinjection (red arrow). **B,** Detection of CTC-driven MBM in 3 mice (circled in yellow) as early as 24 hours following CTC intracardiac injection. These mice were selected for longitudinal MRI MBM imaging (MRI-MBM CDXs). **C,** Parallel pathologic evaluation of CTC-injected mice detecting the presence of MBM along with metastasis to other organs (red arrows), reflecting the target organ metastatic specificity of clinical melanoma. **D,** 3D IVIS tomography of representative CTC MBM mice showing metastatic dissemination, notably to brain (MBM; red arrows). See “Materials and Methods” for experimental details.

As next step, we employed MRI to develop the first CTC-driven, MRI-associated CTC xenograft model (MRI-MBM CDX; Fig. 3). While we were able to consistently detect MBM at 4 weeks following 70W-SM3 cell injection; in one group employing 10 male NSG mice, 3 presented MBM IVIS as early as 24 hours (Fig. 3A and B). Total flux of MBM signal in animal brains was quantified by IVIS and confirmed to be higher in these mice compared with ones without MBM (Supplementary Fig. S2). Accordingly, these animals were selected for sequential MRI, while the remaining 7 mice underwent weekly IVIS imaging parallel to MRI to monitor MBM occurrence and progression. Two mice developed MBM at 4-week point postinjection while another mouse presented with MBM at 8 weeks (Fig. 3A). 3D IVIS virtual tomography was performed to reconstruct brain tumors in 3D with the identification of multiple MBM (Fig. 3D). Mouse necropsies confirmed multiple brain metastatic sites, along with metastatic spread to lungs, liver, stomach, and spleen (Fig. 3C). Because of the high metastatic burden, mice were sacrificed at 8–10 weeks postinjection. However, MBM-IVIS signal specificity for the CTC-derived clone (70W-SM3 cells) was confirmed by parallel analyses employing human melanoma cells (MeWo) which are known to metastasize to lung but not to brain (49). Lung metastasis but no MBM was detected in MeWo-injected animals (Supplementary Fig. S3).

**FIGURE 4:**
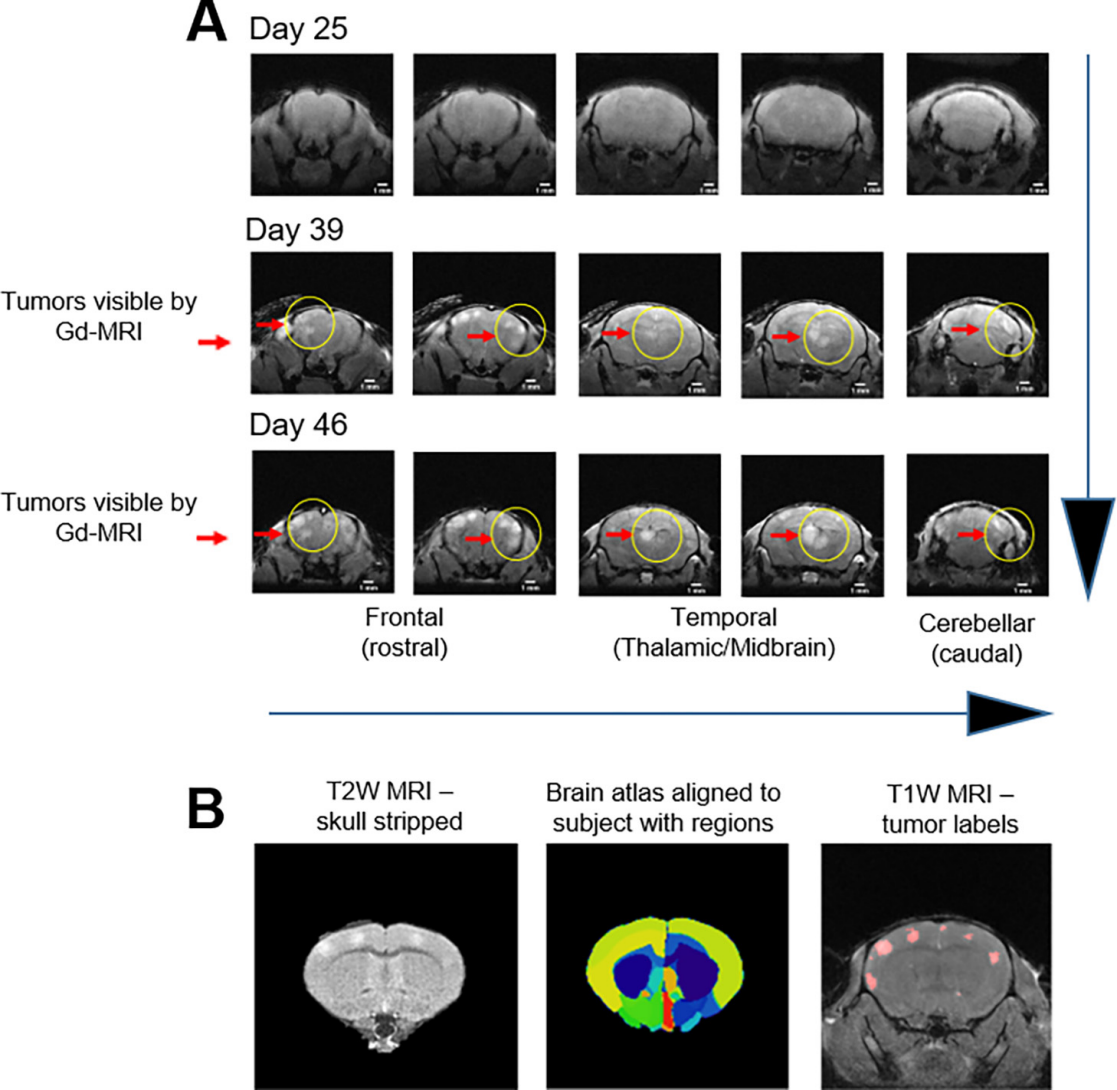
**A,** Spatial and temporal MBM onset by MRI analyses using CDX mice (MRI-MBM CDXs). MRI-MBM CDXs underwent MRI analyses biweekly employing the Bruker 7-Tesla PET/MRI scanner. While no MBM was found at day 25 post-CTC injection, MRI detected the presence of MBM in all CDX mice at subsequent timepoints (day 39, day 46 after CTC injection) with specific MBM localization in the FL, PTL, and cerebellum regions (red arrows, yellow circles), reflecting the MBM presentation in patients. **B,** Spatial and temporal MRI analytic quantitation of MRI-MBM CDXs. Representative images of CTC-MBM CDXs employing the skull stripping procedure for removal of extra brain tissue to visualize brain tumors (left), brain atlas based MBM assessment showing alignment to 62 brain regions using ANTs Python program (middle), or T1W MRI displaying MBM sizes generated by the 3D Slicer software program (right). See “Materials and Methods” for experimental details.

**FIGURE 5:**
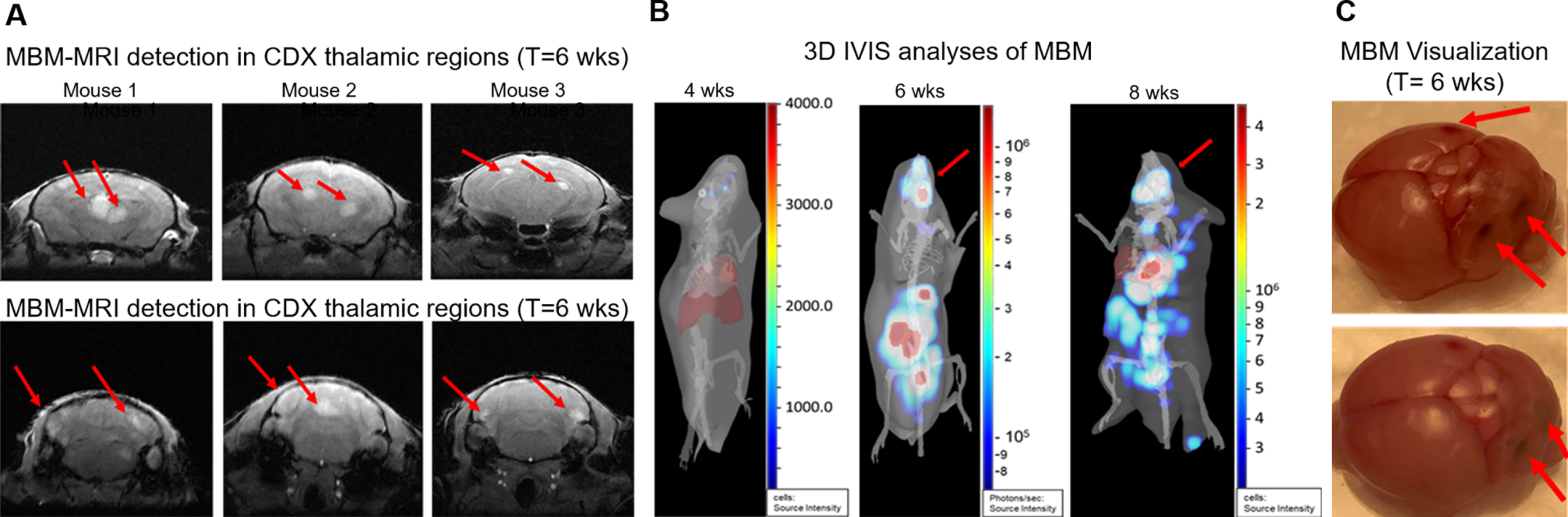
Spatial and temporal MRI-MBM analyses of CDXs along with pathological assessment. The MRI-MBM detection in thalamic regions of the temporal and cerebellar regions of MRI-MBM CDXs was confirmed by 3D IVIS tomography showing MBM progression overtime (4–8 weeks, red arrows; **A**) and by pathologic evaluation for MBM presence in mice brain necropsies following MRI (**B**). Representative mouse brains with MBM (red arrows) are shown (**C**). See “Materials and Methods” for experimental details.

Longitudinal MRI (Fig. 4) was performed biweekly to monitor MBM progression and to determine any ensuing MBM. MRI was carried out using the advanced 7-Tesla MRI scanner with high signal-to-noise ratio, translating into enhanced resolution and improved differentiation among brain tissue (50). No brain masses were visible by MRI by the third timepoint (25 days postinjection; Fig. 4A); however, MBM was MRI detectable at day 39 postinjection in all 3 animals (Fig. 4B and 5). Importantly, tumors localized to specific regions of the brain—FL, PTL, and cerebellum—which recapitulated MBM clinical presentation (Fig. 5A), validating our MRI-MBM CDX model for CTC MBM regional specificity (Fig. 5C). Longitudinal 3D IVIS tomography was executed to reconstruct brain tumor development in 3D over the period of 8 weeks (Fig. 5B).

Furthermore, MRI-detectable tumor volume was quantified for each region and animal, with FL having the highest tumor burden (Supplementary Table S2). Sequential MRI at day 46 postinjection showed a significant increase of tumor mass in all MBM sites (Fig. 4A; Supplementary Table S2A). Moreover, the average value in tumor volume was calculated by brain region from day 39 to day 46 postinjection (Supplementary Table S2B). The highest values in brain tumor volume were observed in FL, followed by cerebellum and PTL. It was complemented by employing the brain atlas with 62 brain regions normalized to T2W images using ANTs Python, and segmented CE-T1W MRI was implemented to quantify brain tumor volume (Supplementary Table S2B). Negative controls consisted of performing MRI of mice without IVIS-detectable MBM, confirming no MRI-MBM detection (Supplementary Fig. S4).

### Longitudinal CDX CTC Levels are MBM Dependent

**FIGURE 6:**
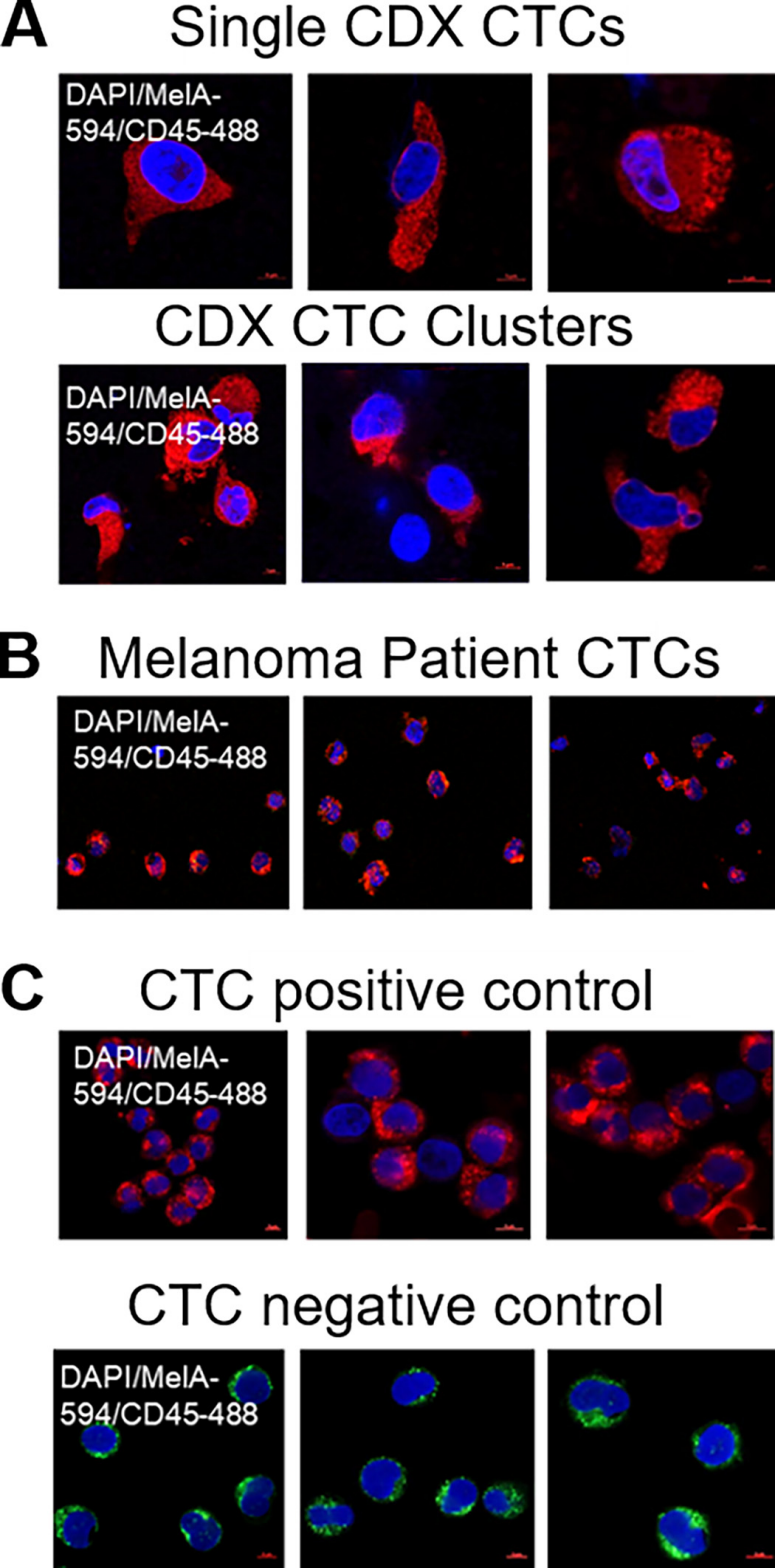
The capture and interrogation of CTCs from CDXs using the CTC Parsortix platform. Representative images of human melanoma CTCs captured/visualized by the CTC Parsortix platform, either as *ex vivo* single CTCs or homotypic CTC clusters from blood of MRI-MBM mice (*N* = 3; **A**), patients with MBM (*N* = 3; **B**), or as CTC-derived clonal cells (70W-SM3) spiked (positive control) in blood from healthy donors (negative control; *N* = 3; **C**). CTCs were defined for absence of human FITC-CD45 (green fluorescence); however, presence of human Melan-A/Alexa Fluor 594 (red fluorescence), and DAPI staining within the separation Parsortix cassette. Human Melan-A^+^/DAPI^+^/CD45^−^ cells were then visualized and quantitated by confocal Zeiss LSM800 microscopy. See “Materials and Methods” for experimental details.

To determine the correlation between MRI-MBM and CTC content in our CDX model, we captured and interrogated CTCs from MBM/No MBM mice longitudinally by retro-orbital blood (150 μL) collection. Blood from three MRI-MBM CDXs was combined following each blood draw and analyzed by the CTC Parsortix microfluidic device to capture single CTCs and CTC clusters based upon their size and deformability. Parsortix-captured CTCs were immunostained for human Mel-A Alexa Fluor 594, human FITC-CD45, and DAPI (markers have been used to define human melanoma CTCs as Mel-A^+^/DAPI^+^/CD45^−^ cells; refs. 25, 28) within the Parsortix separation cassette, visualized and counted (Fig. 6A). Interestingly, while CTCs were not detected in murine blood for the first 4 weeks (Supplementary Table S3), CTCs could be captured at 6 weeks, and this correlated with the MRI-MBM detection in these animals (Fig. 6A). Second, considerable increase of CTC numbers was observed at 8 weeks postinjection, when the number of single CTCs increased 4-fold. Third, homotypic CTC clusters were also detected at this time, either small (2, 3, 4 cells) or large (5 cells or greater) which are pivotal since they have stronger metastatic potential and higher resistance against therapy than single CTCs (refs. 51, 52; Fig. 6; Supplementary Table S3B). These findings were also consistent with the increase of brain tumor burden in these animals at the last MRI timepoint, suggesting that growing MBM promoted shedding of higher CTC numbers into the bloodstream of MRI-MBM CDXs, and confirmed the severity of MRI temporal and spatial detection (Fig. 4). These results were complemented by multiple Parsortix CTC analyses involving: (i) No MBM CDXs (but with metastasis to other organs) which showed detection of CTCs at 6 weeks; however, no significant increase in CTC number or presence of CTC clusters were observed in 2 weeks (Supplementary Table S3C); (ii) metastatic but with no MBM patient blood samples which correlated with the above findings, for example, a patient possessing high number of single CTCs (77 CTCs per 100 μL of blood; Fig. 6B; Supplementary Table S3A); (iii) blood from healthy donors spiked with CTC-derived clonal cells at increasing concentrations which were correlative with increasing numbers of CTCs; (iv) healthy donors’ blood resulting in no CTC detection (Fig. 6C).

### The Multilevel CTC Transcriptomic Characterization of MBM CDXs

**FIGURE 7:**
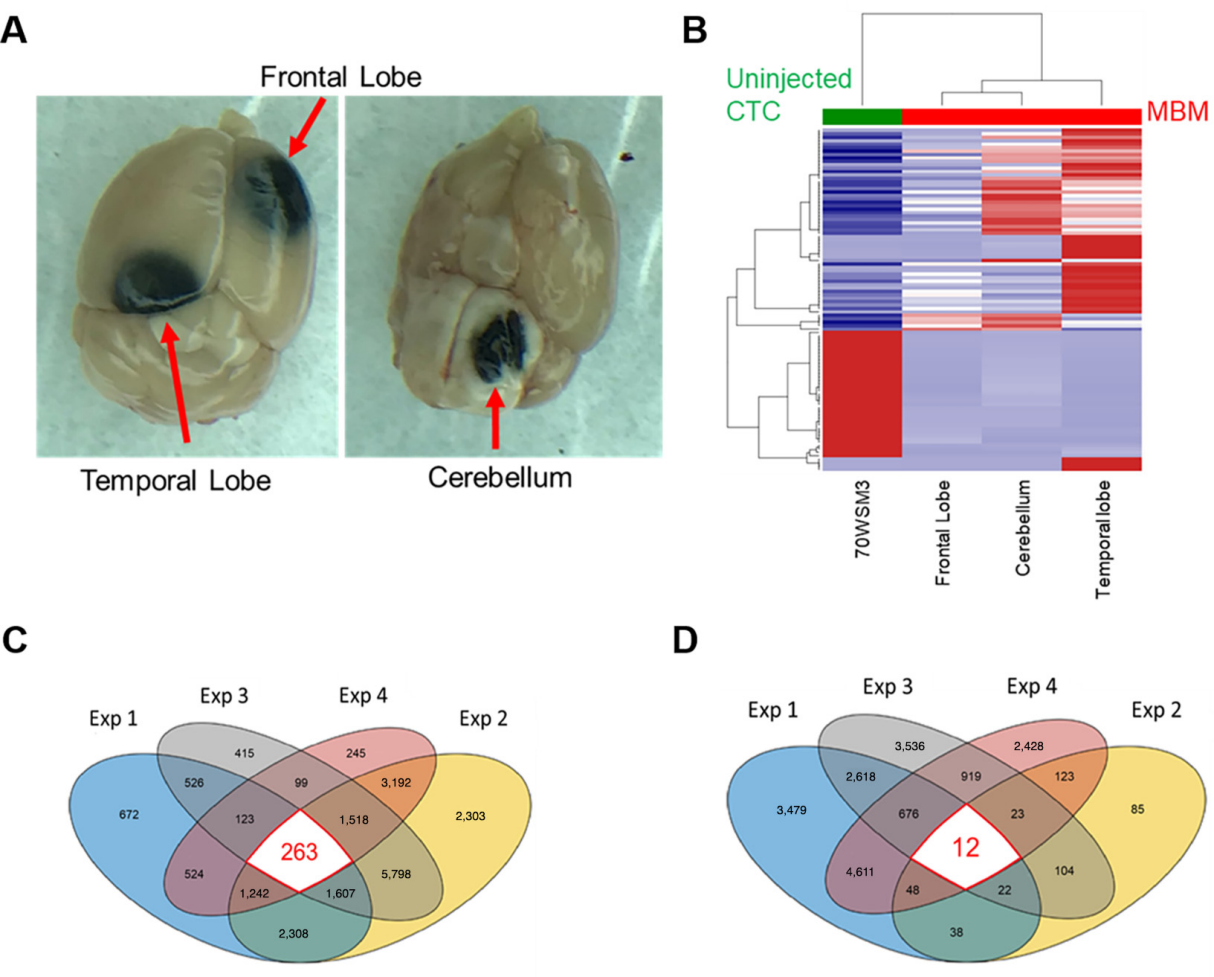
The hierarchical transcriptional classification of CTC-driven MBM. Regional specificity of CTC-driven MBM was detected in FL, temporal lobe, and cerebellum regions of CDXs (**A**), with a distinct MBM region–dependent transcriptional profiling/hierarchical clustering displaying unique gene expression patterns compared with uninjected CTC-derived clonal cell (70W-SM3-Luc2; **B**). Venn diagrams showing 263 upregulated (**C**) and 12 downregulated (**D**) genes as result of combinatorial gene expression analyses employing a four-pronged experimental approach consisting of transcriptome analyses of: (1) CTCs from MBM versus No MBM CDXs, (2) region-specific CTC MBM versus uninjected CTC-derived clonal cells, (3) LinN cells from MBM versus metastatic/primary patients, and (4) LinN cells longitudinally (0, 3, 6 months) isolated from a patient with MBM. See “Materials and Methods” for experimental details.

Analyses of gene expression patterns in patients with MBM and patients without MBM indicated distinct differences in their clustering patterns (Fig. 2C). These differences prompted us to investigate variability of gene expression levels in blood of CDX mice with MBM versus animals without MBM. MRI-MBM CDXs mice could not be used in these analyses because CTCs were captured and/or immunostained within the Parsortix cassette, and therefore not accessible to further investigations. Experiments were conducted involving the injection of NSG mice with the highly brain-metastatic CTC-derived clone (5 × 10^5^ cells/mouse, 6 mice/subgroup), monitoring metastatic development by weekly IVIS imaging. Augmented tumor burden was detected over a period of 8 weeks (2 mice developed MBM), afterward necropsies of MBM mice were performed to identify specific MBM sites and blood was collected. Blood samples were then analyzed by Parsortix (no immunostaining) to harvest CTCs for RNA-seq interrogation. Conversely, the remaining 4 mice developed metastasis to other organs, for example, liver, spleen, etc., but not to brain, and were similarly processed. Consistent with MRI findings, MBM CDXs developed tumors in FL, PTL, and cerebellum (Fig. 7A). Single-cell RNA-seq was executed to compare gene expression levels in different regions of the brain, with libraries aligned to the human and not mouse genome. Resulting heatmaps displayed significant variation among brain regions, with distinct patterns which were significantly different from uninjected CTC-derived clonal cells (Fig. 7B). Results suggest that changes in molecular pathways occur upon the successful CTC MBM onset are region specific.

### The Identification of the CTC RPL/RPS Gene Signature by Multilevel MBM Discrimination

**FIGURE 8:**
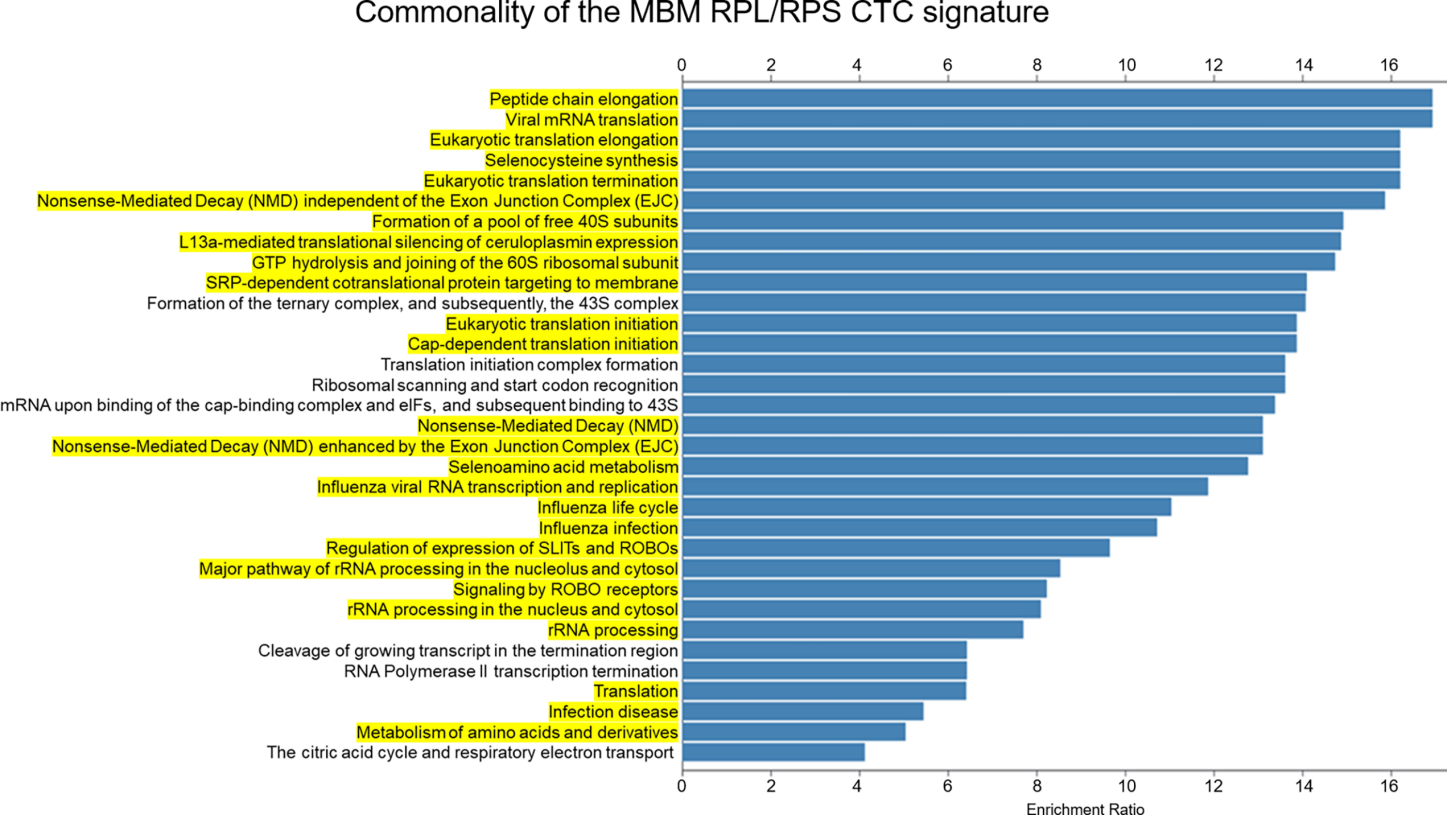
The CTC RPL/RPS gene pathways of MBM. List of the top molecular pathways resulting from the four-pronged experimental approach and hierarchal clustering of MBM samples (Reactome pathway database). Highlighted in yellow are CTC translational pathways containing the CTC RPL/RPS gene signature of MBM. See “Materials and Methods” for experimental details.

To identify a unique CTC genetic signature associated with MBM, we performed bioinformatics analyses involving unsupervised transcriptomic profiling of MBM detected in patients and animal samples, employing a four-pronged approach to identify a common CTC MBM signature. Specifically, this consisted in CTC gene expression analyses involving: (i) primary, metastatic (No MBM), and patients with MBM, (ii) CTC longitudinal profiling (9 months period) in a patient diagnosed with MBM; (iii) blood from MBM/No MBM CDXs; and (iv) MBM CDX tissues spatially distinct (FL, PTL, and cerebellum). Transcriptomes were mapped and/or analyzed altogether to yield 263 common upregulated and 12 downregulated genes of MBM (Fig. 7B and D, respectively). Furthermore, reactome analyses against the hallmark gene sets generated a list of statistically significant pathways involved in MBM onset and progression (Fig. 8). Notably, 26 of 33 gene pathways had 21 commonly shared genes (Supplementary Table S4), with all these genes being members of the large or small ribosomal proteins (RPL/RPS) gene families and involved in translational processes: the CTC RPL/RPS gene signature of MBM (Fig. 8—highlighted in yellow). Of note, nine RPS common genes were shared among higher number of pathways and were found in 30 of 33 pathways. Furthermore, RPL/RPS genes were highly significant in multilevel analyses: the top 20 genes out of 263 total upregulated genes included nine RPL/RPS-related genes (Supplementary Table S5). Equally relevant, patients with primary melanoma and metastatic patients with No MBM (Fig. 2) did not possess high RPL/RPS gene expression markers, in striking contrast from patients diagnosed with MBM: mean RPL/RPS values in patients with MBM had 2- to 10-fold increase in the level of ribosomal proteins, compared with patients with No MBM (Supplementary Table S6). Significantly elevated RPL/RPS expression was also detected in most molecular pathways involved in translational programs known of fundamental importance in cancer progression (Fig. 8; refs. 22, 53).

## Discussion

This study centered on investigating the biology of CTCs associated with the onset and progression of MBM, and provides first-time evidence of a specific CTC gene signature (“The CTC RPL/RPS gene signature”) associated with MBM. This was achieved by multilevel analyses, employing a novel MRI-dependent MBM CDX model, the gene expression interrogation of CTCs/Lin− cell populations isolated from patients at distinct stages of disease progression (primary, metastatic melanoma diagnosed with or without MBM), CTC longitudinal monitoring (patient diagnosed with MBM), or by the interrogation of CDX MBM evaluated spatially or temporally. Our multilevel approach included comparing blood samples of metastatic patients with brain metastasis (MBM) versus metastatic patients with tumor cell dissemination to non-brain distant sites, for example, lungs, but not to brain (No MBM). The discovery of the CTC RPL/RPS gene signature of MBM has relevance because variability in ribosomal composition may result in the generation of a “onco-ribosome” which drives increased translation, cell proliferation, and tumorigenesis by means of modulating oncogenic signaling pathways (23, 54). Enhanced ribosome biogenesis may be critical in achieving metabolic plasticity (22).

Melanoma is the most aggressive skin cancer whose rate of diagnosis is advancing faster than any other cancer type of cancer, due to melanoma's proclivity to metastasize throughout the body. Specifically, MBM significantly reduces overall survival and is linked to poor clinical outcomes, representing a significant biological and clinical challenge (1, 5, 6, 8, 9). One of the fundamental questions still unanswered in the melanoma field is to characterize metastatic-competent CTCs. In contrast to the majority of CTC investigations, we employed a multilevel approach, temporal and spatial, to derive insights for the key CTC properties responsible for overt MBM. We demonstrated that transcriptional subtyping of melanoma CTCs resulted in the common CTC RPL/RPS gene signature, possibly responsible for MBM onset and progression. We show that transcriptional subtyping of CTCs from the Lin− cell population of patients with MBM provided distinct genetic signatures. Meanwhile, CTCs from patients with primary melanoma or patients with melanoma with metastasis to non-brain organs did not share MBM transcriptional profiling. In addition, we performed the first longitudinal CTC transcriptomic analyses of a patient with MBM over a period of 6 months (Fig. 3D). These transcriptomic analyses were pivotal in identifying the CTC RPL/RPS gene signature of MBM. To further evaluate this signature, additional multilevel studies were performed using MRI CTC-driven mouse model.

Currently, there is a paucity of experimental models of brain metastasis due to inefficient brain colonization, disease latency, and early animal mortality due to metastatic burden in other organs (1, 8). Although these models have been an invaluable tool to study MBM, the process by which they have been generated varies greatly from one occurring in patients and involving CTCs. We report establishing the first successful MRI CTC-driven xenograft model of MBM (MRI-MBM CDX model) which mimics human disease development (Figs. 4 and 5). MRI is a noninvasive imaging technique that has been considered the gold standard for MBM identification, evaluation of clinical brain metastasis, and response to therapy in these settings (55). Importantly, MRI can be used for the longitudinal screening of disease progression within the same individual. Our experimental model allowed us to detect and investigate MBM 24 hours postinjection. This model provides the advantage of performing comprehensive analysis of the multistep process of brain metastasis using a CTC-derived clone (70W-SM3 cells). Longitudinal MRI screening of MBM mice resulted in the identification of specific sites of brain colonization; FL, PTL, and cerebellum, confirming to be major MBM niches as seen by routine radiologic imaging. We carried out detailed transcriptomic analysis of the brain tumors from FL, PTL, and cerebellum to interrogate MBM-CTC specificity.

A number of recent studies have reported a link between abnormal ribosome synthesis and malignancy formation (22–25). A study reported that dysregulation of translation in a breast cancer study has been linked to increased metastasis (24). Specifically, increase of RPL15 expression triggered massive metastatic spread to distant organs and induced translation of other core ribosomal subunits. Also, dysregulation in ribosome biogenesis has been linked to increased tumor burden (22). Thus, enhanced expression of ribosomal proteins could potentially result in ribosomopathies associated with MBM development and progression (22, 23). Of note, a recent study has demonstrated that increased tumor-specific total mRNA expression (TmS) is observed in 6,580 patient tumors across 15 cancer types and is correlated to disease progression and reduced overall survival. Quantification of cell-type specific total mRNA transcripts can be a prognostic factor in the systemic evaluation of patients to predict cancer progression and clinical outcomes, with TmS expression reported to be an indicator of phenotypic plasticity (53). To the best of our knowledge, this is the first study to identify a common CTC RPL/RPS genetic signature of MBM using multilevel analyses that could be used in future therapeutic applications.

In synchrony with the above findings and collectively, our study suggests that the cell translational machine may have another layer of regulation of gene expression refining CTC-associated prognostication. Ribosome biogenesis is a highly coordinated process between RPL/RPS proteins and rRNA assembly factors. This implies a specific vulnerability of CTCs and suggests the targeting of ribosomal biogenesis significantly affects CTC metastatic states. As a way to suppress aggressive CTC subsets which are characterized by high RPL/RPS content, genetic screening of ribosomal protein expression in patients with MBM could potentially be a prognostic factor of the disease severity and outcomes.

This study has some limitations. First, it is based on a limited number of patients with melanoma; therefore, we cannot conclude that all patients with MBM follow these gene pathways and CTC signature. The expected presence of heterogeneity and cancer subtypes among patients adds complexity to drawing definitive conclusions. Second, the animal models had a small sample size and cannot eliminate the possibility of an inherent sampling bias. Third, we cannot exclude the possibility that the CTC RPL/RPS gene signature can lead to altered extraribosomal functions (56). Fourth, a limitation of the study includes the use of a single MBM CTC-derived clone in the majority of the experiments due to the laborious, tedious, and time-consuming work of establishing a MBM CTC clone that successfully recapitulated MBM development and progression in patients with melanoma. Similarly, the longitudinal study was performed on a single MBM patient due to the limited samples availability, patients’ consent to these analyses, or patients’ poor survival due to MBM diagnosis and progression. Finally, there might be additional parallel pathways driving or contributing to MBM that were not detected or evaluated in these analyses. Additional studies are needed to address these limitations. Of note, we counteract these limitations with the novel analysis that emphasizes the role of RPL/RPS CTC signature in relation to brain metastasis, regardless of cancer type. The RPL/RPS signature of brain metastasis was not observed exclusively in melanoma; 19 RPL/RPS genes of the MBM CTC signature (out of 21) were shared between brain metastasis of melanoma and breast cancer, latter by literature searches of reports investigating brain-homing breast cancer cell lines (57). Our approach can be viewed as a novel analysis of MBM using a four-level discrimination to provide a relevant and clinically meaningful gene signature. In conclusion, the identification of the melanoma CTC RPL/RPS gene signature, common to all MBM samples analyzed, can drive the hyperactivation of ribosomal biogenesis and aid MBM onset and progression. These findings provide the conduit for translation to the clinic and set the stage for the development of therapeutic agents to improve melanoma patient care, notably MBM.

## Supplementary Material

Supplementary Data S1 Supplemental figures and tables
