# Supplementary material for: The RPL/RPS Gene Signature of Melanoma CTCs Associates with Brain Metastasis
Source: Cancer Res Commun. 2022 Nov 16;2(11):1436–48. doi: 10.1158/2767-9764.CRC-22-0337 (PMC9668078; doi:10.1158/2767-9764.CRC-22-0337)
Supplement: Supplementary Data S1 — Supplemental figures and tables [file crc-22-0337-s01.docx]

# Figure S1


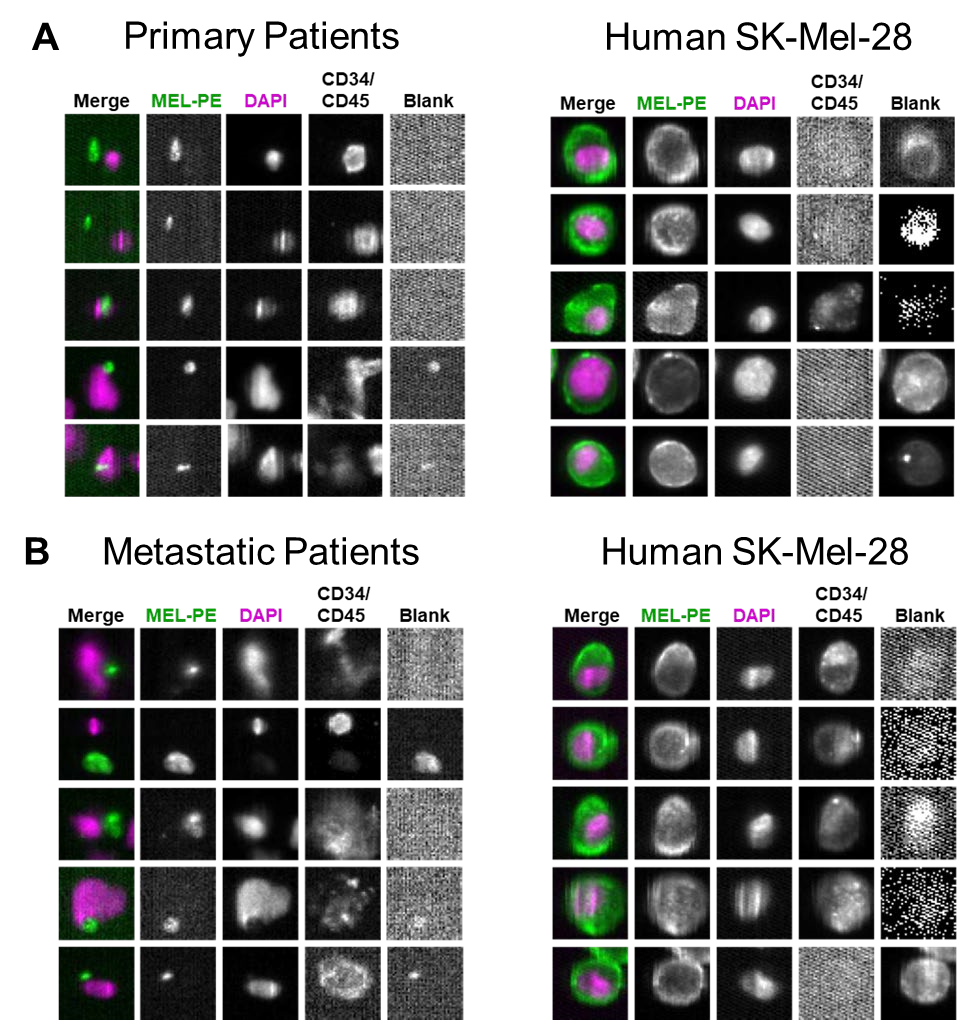


## Supplementary Fig. 1

CellSearch analyses of blood from healthy donors (normal blood), melanoma CTCderived clone 70W-SM3 spiked in blood, and human melanoma SK-Mel-28 cells. Normal blood from healthy donors was processed using CellSearch (upper left panel). No melanoma CTCs (MEL-PE^+^/DAPI^+^/CD45^-^ cells) were captured. Spiked melanoma CTCderived clone (70W-SM3 cells)(lower left panels) and human melanoma SK-Mel-28 cells were used as respective positive controls (right panels) used as a positive control. Displayed are the original CellSearch images using CellBrowser^TM^ software (10x magnification).

# Figure S2


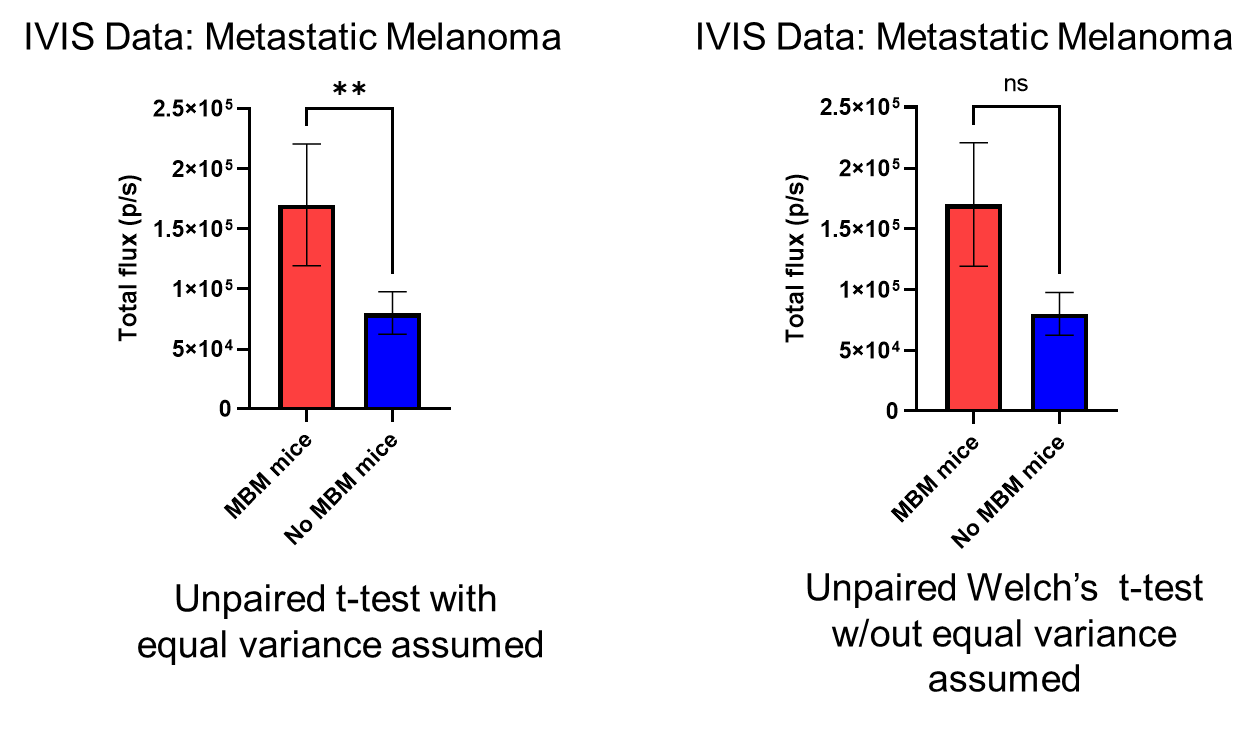


## Supplementary Fig. 2

Quantitation of IVIS analyses in CTC-derived clone-injected NGS mice. Total flux of the mouse brain region was measured by IVIS imaging system 24 hours following injection of CTC-Derived clonal cells (70W-SM3). Mice having MBM were subsequently processed were sent for MRI imaging (N=3), while mice with No MBM were subjected to IVIS imaging (N=7).

# Figure S3


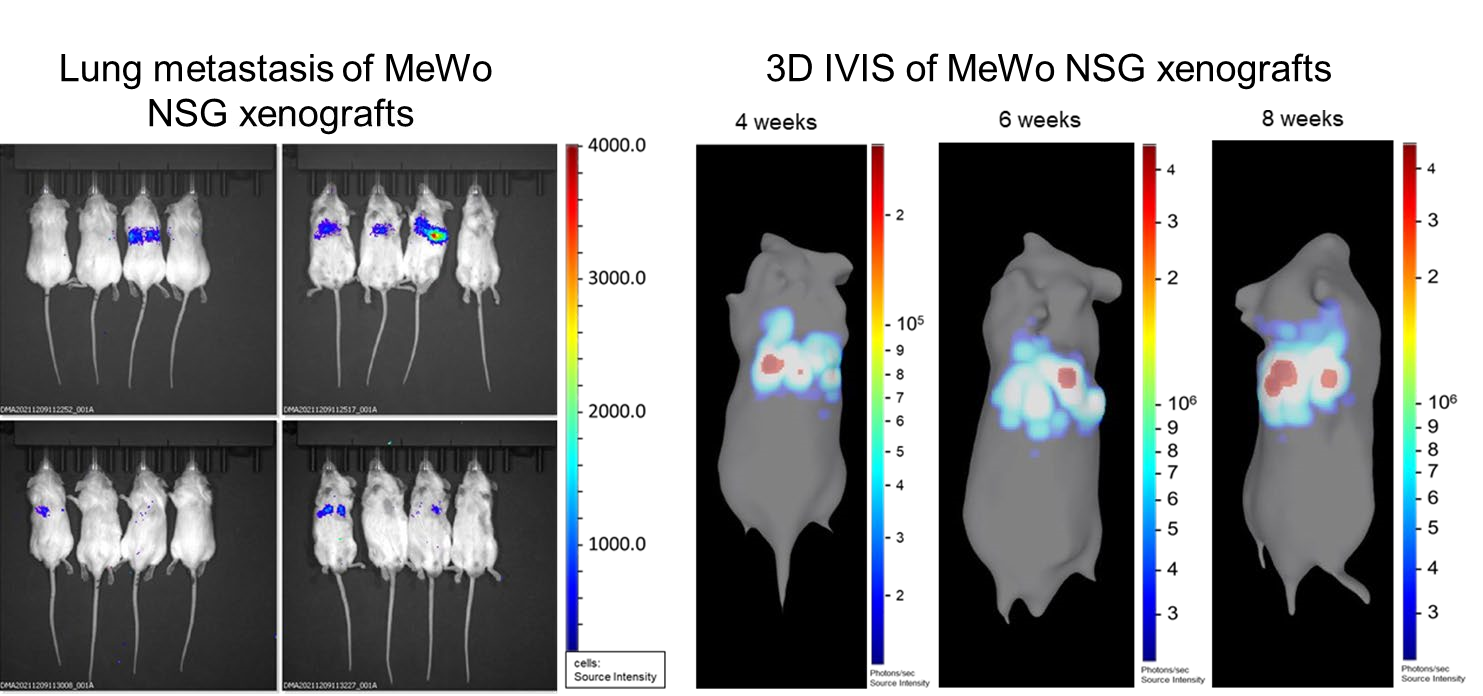


## Supplementary Fig. 3

Lung-targeting xenograft model of melanoma. Six NSG mice were injected with human melanoma cells (5.0 x 10E5 MeWo-Luc2 cells) and imaged by IVIS 24 hours later. No brain metastasis was detected in these mice (left panel). 3D IVIS tomography was performed biweekly to evaluate metastatic patterns in the animals.

# Figure S4


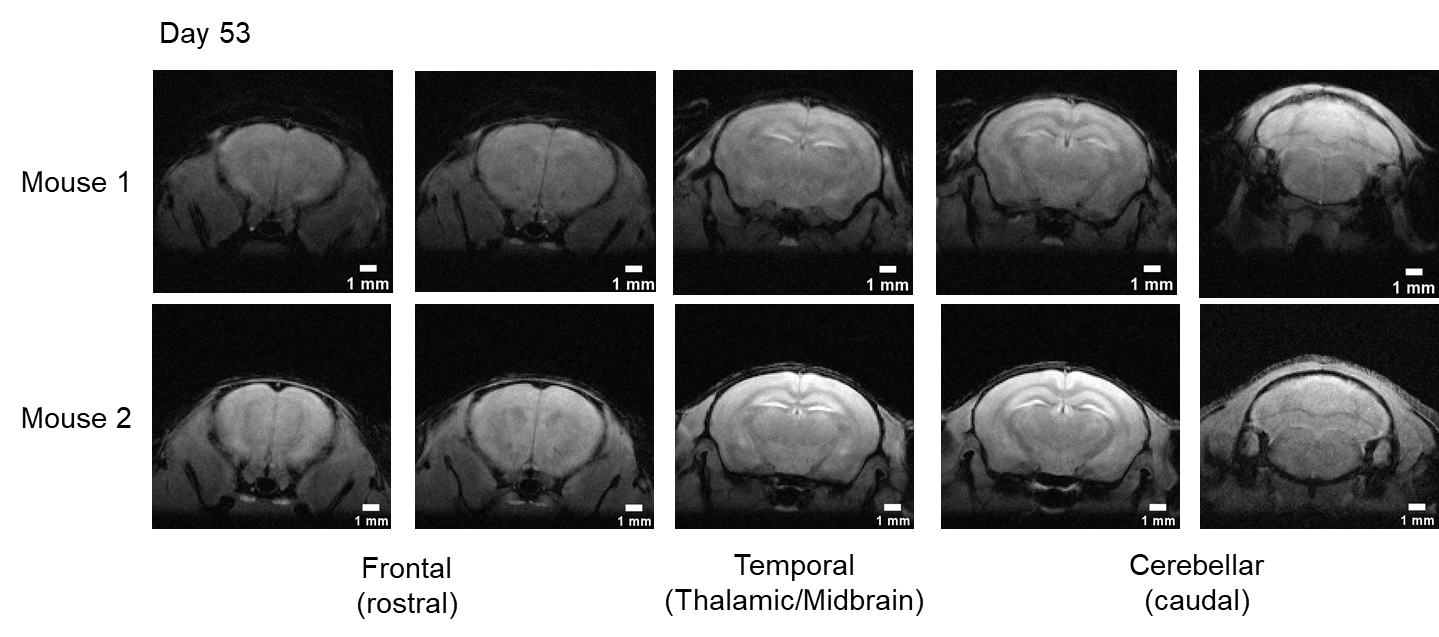


## Supplementary Fig. 4

MRI imaging of female mice without MBM. Four NSG mice were injected with CTCderived clonal cells (5.0 x 10E5 70W-SM3-Luc2 cells) and processed for MRI imaging.

MRI was performed biweekly using manganese contrast agent. No MBM were detected.

# Table S1


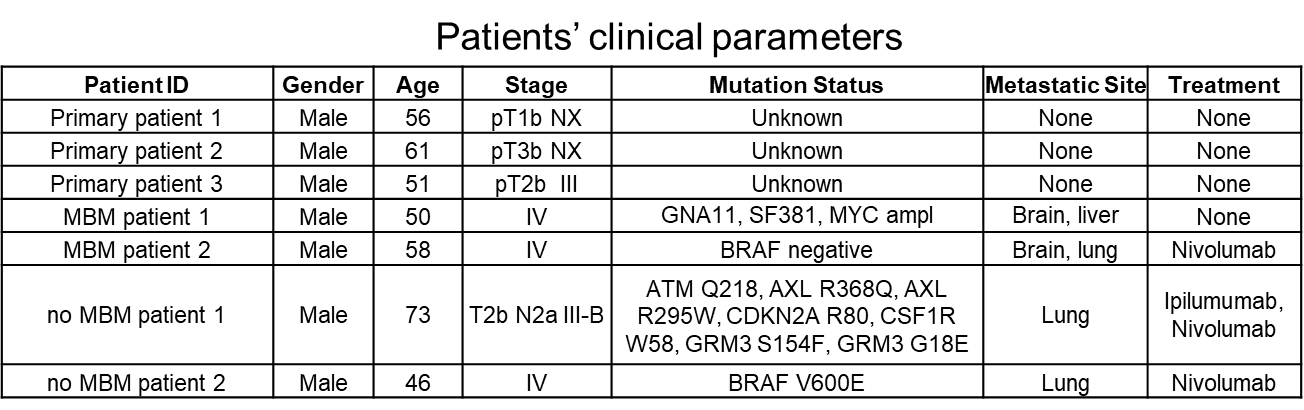


**Supplementary Table 1**.

Demographics and clinical-pathological characteristics of melanoma patients of this study. Clinical parameters of patients include gender, age, stage, mutation status, metastatic site, and treatment.

Table S2


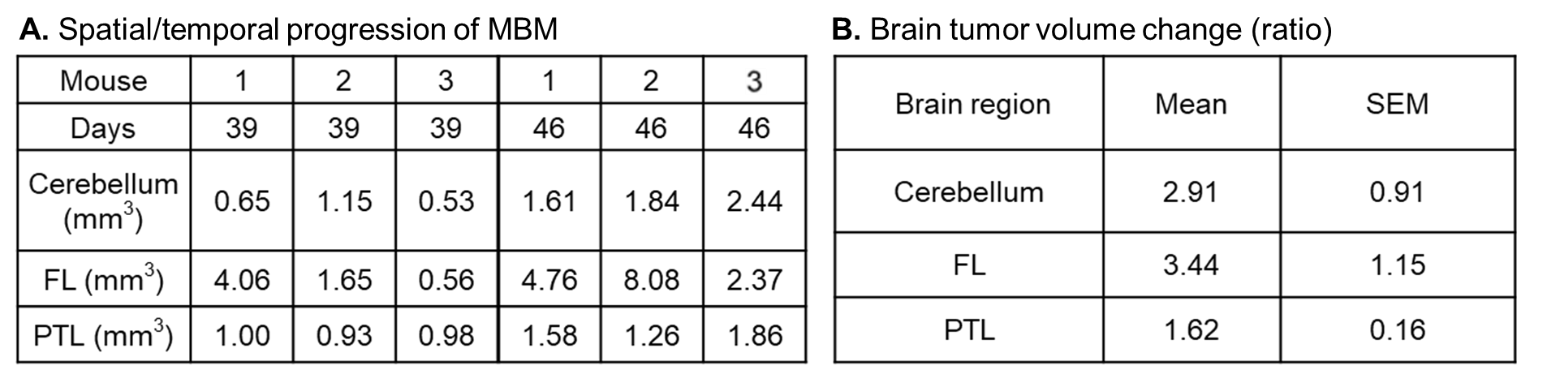


**Supplementary Table S2**.

Spatial and temporal growth of MBM. Table **A** shows analyses of spatial and temporal MRI-MBM progression over time in various brain regions (FL = Frontal Lobe; PTL = Parietotemporal Lobe). MBM volume/ratios and statistical validation (SEM) are presented in Table **B**. See “Materials and Methods” for experimental details.

Table S3


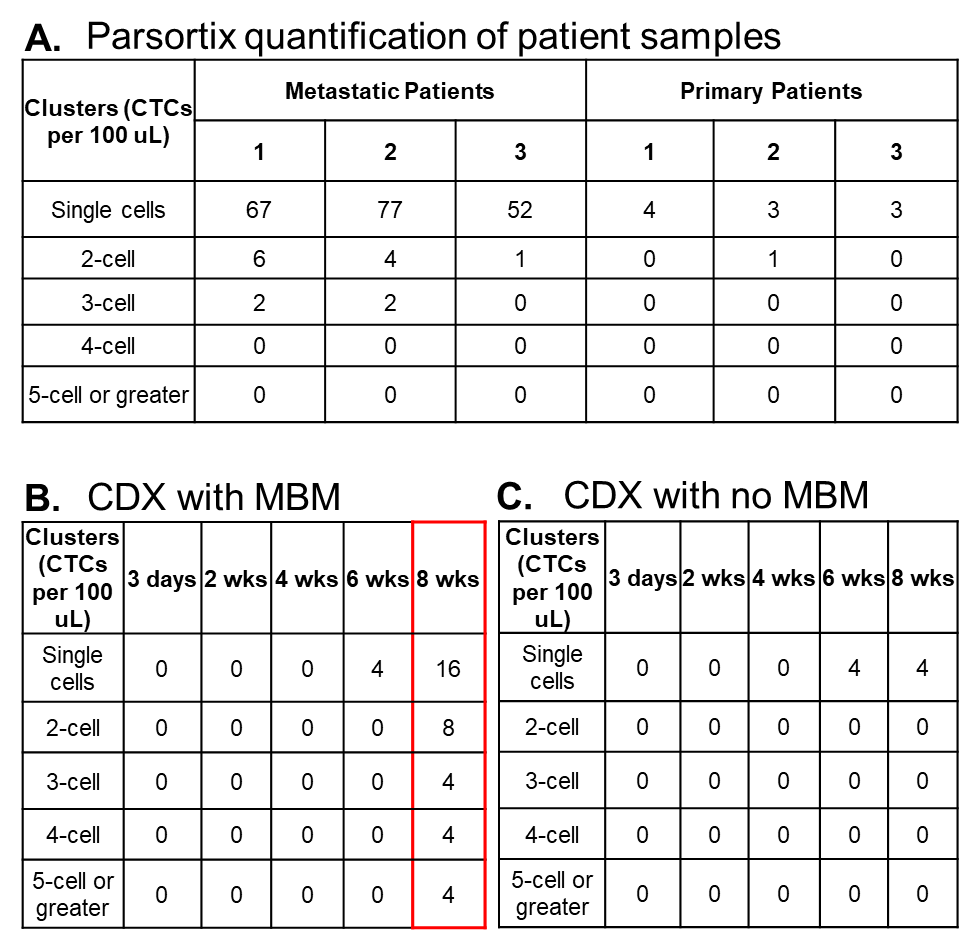


**Supplementary Table 3**.

Enumeration of CTCs captured by Parsortix. **A**, Quantitation of CTCs from metastatic melanoma patients not diagnosed with MBM (No MBM). Higher CTC numbers were captured and visualized by the CTC Parsortix platform in MBM (**B**) vs No MBM CDXs (**C**) over time and consistent with MRI-MBM/pathological detection. See “Materials and Methods” for experimental details.

Table S4


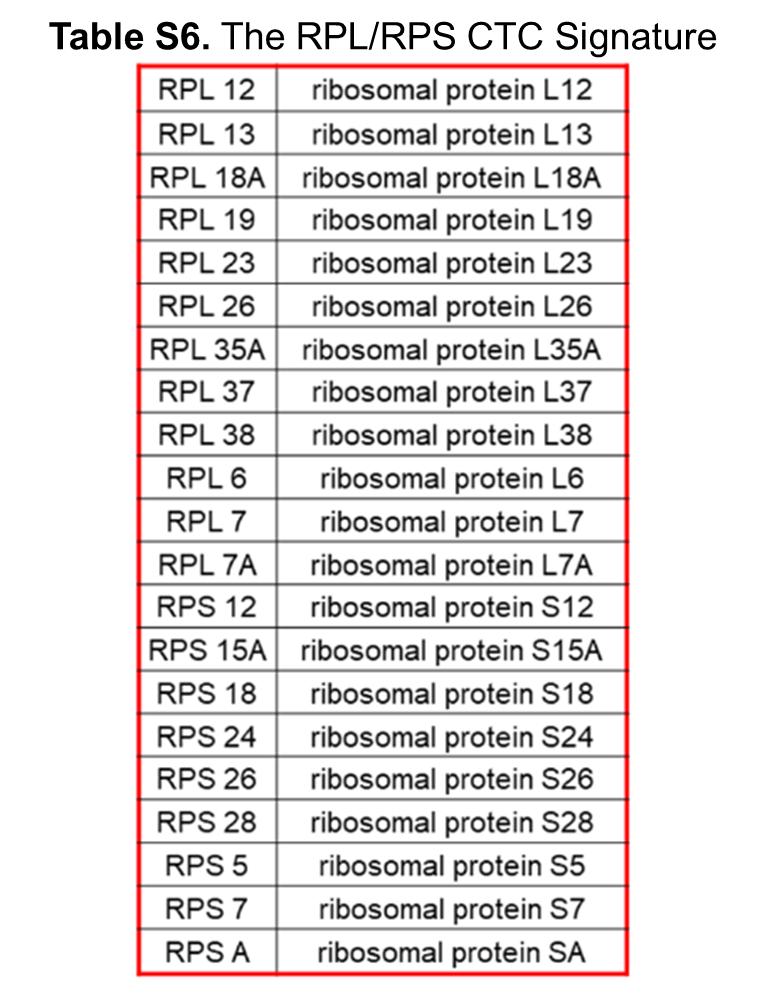


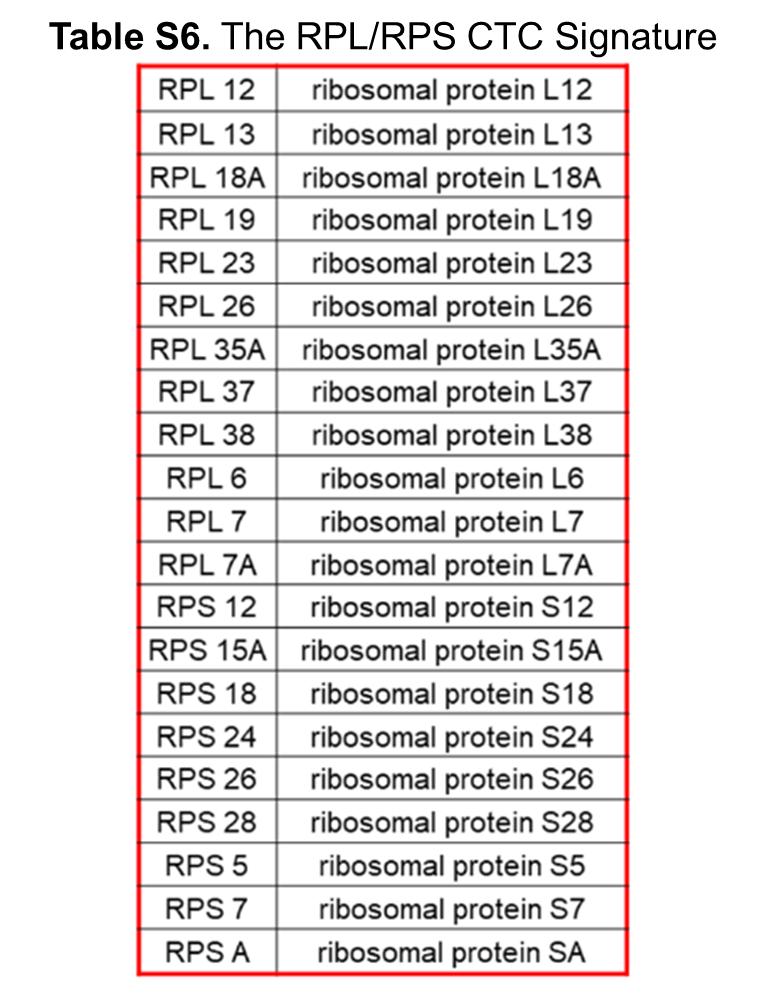


**Supplementary Table 4**.

The CTC RPL/RPS gene signature of MBM. Table shows the RPL/RPS CTC gene signature as result of the four-pronged hierarchical clustering among all samples and translational pathways analyzed (Reactome pathway database). The 21 RPS/RPL genes of the commonly-shared CTC gene signature of MBM are listed.

# Table S5


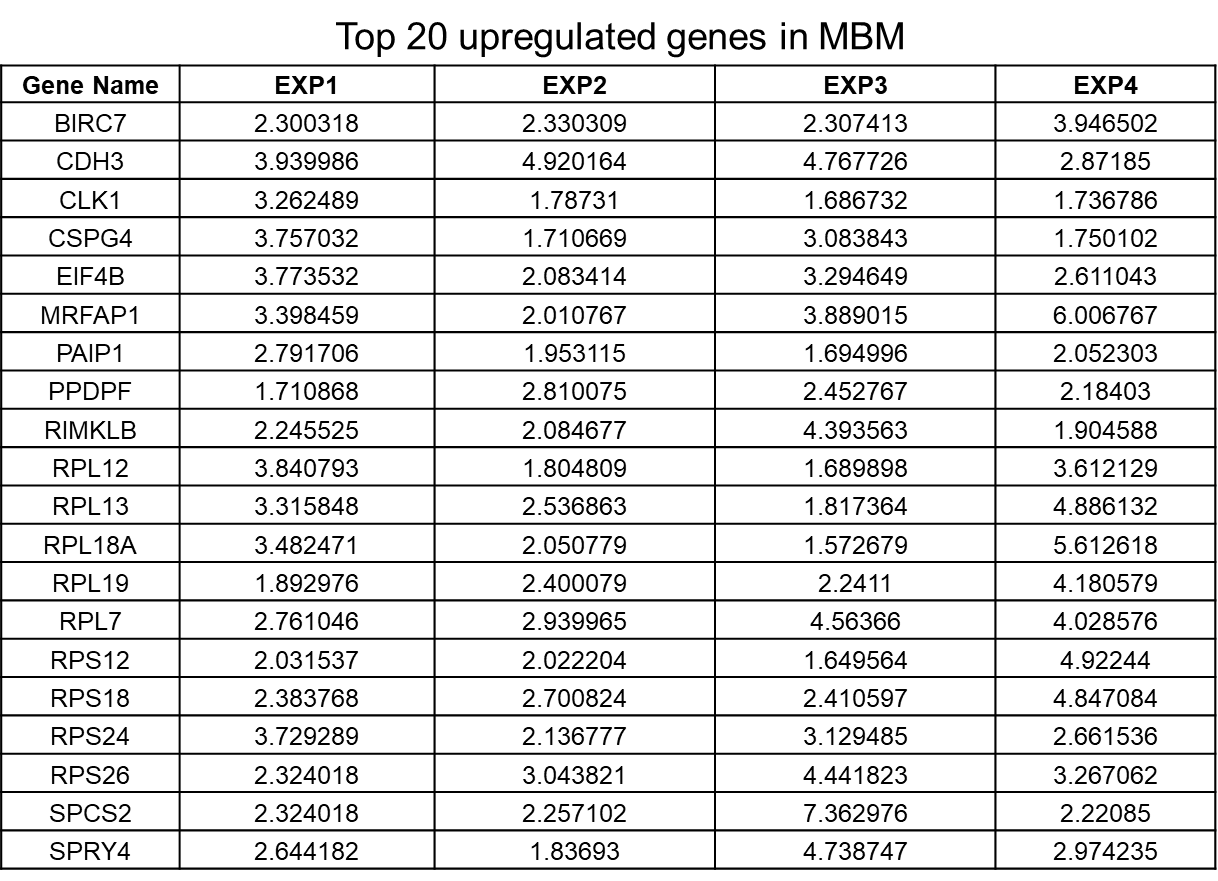


**Supplementary Table 5.**

Top 20 upregulated genes in MBM by the four-pronged experimental approach used in this study. Nine out of 20 upregulated genes are RPL/RPS genes of the MBM CTC signature.

# Table S6


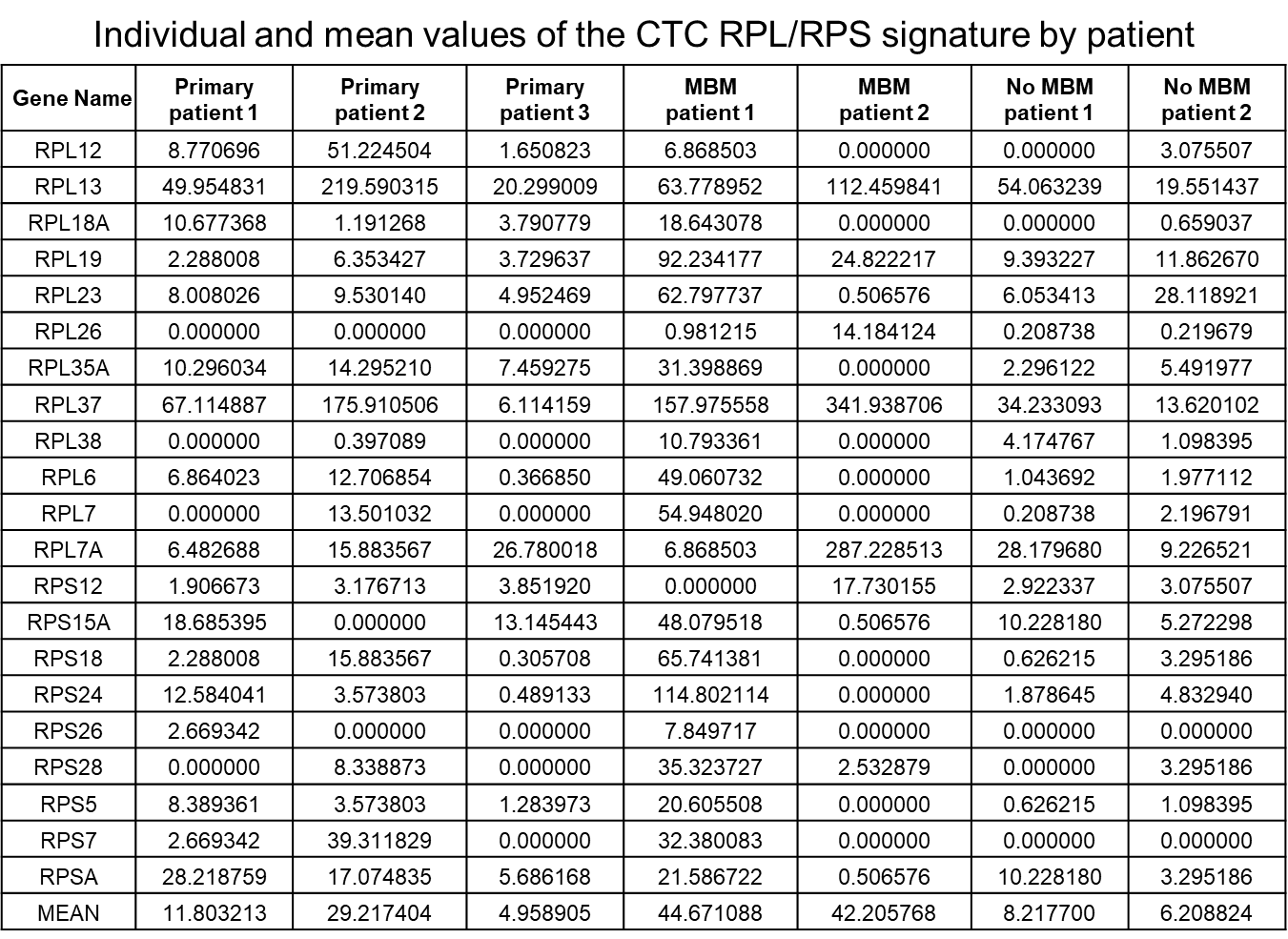


**Supplementary Table 6**.

Individual and mean values (cpm) of RPL/RPS CTC MBM signature per patient analyzed. MBM patients showed higher mean values of RPL/RPS genes vs patients with No MBM.
